# The divergent cts1 type VI secretion system locus of Citrobacter rodentium is controlled by multiple promoters that are silenced by H-NS, with CpxR contributing to their activation upon derepression

**DOI:** 10.1099/mic.0.001756

**Published:** 2026-08-12

**Authors:** Stephanie Ortiz-Jiménez, Diana Sabina Carrasco-Navarro, Jose L. Puente

**Affiliations:** 1 Departamento de Microbiología Molecular, Instituto de Biotecnología, Universidad Nacional Autónoma de México, Cuernavaca, Morelos, México

**Keywords:** *Citrobacter rodentium*, CpxR, H-NS, internal promoters, T6SS, transcriptional regulation

## Abstract

The type VI secretion system (T6SS) is a molecular nanomachine that provides Gram-negative bacteria with competitive advantages during host colonization and interbacterial interactions. *Citrobacter rodentium*, a mouse-specific attaching and effacing pathogen, employs a T6SS encoded by the *cts1* locus to outcompete the gut microbiota and establish infection. However, under standard laboratory growth conditions, *cts1* expression remains silent and the regulatory mechanisms governing its activation are unknown. Here, we investigated the regulation of *cts1,* which is organized into two divergent gene clusters, *cts1L* and *cts1R*. Transcriptional fusions of the *cts1L-cts1R* intergenic region in both orientations were inactive in wild-type *C. rodentium,* but became active in the absence of the global regulator H-NS. Systematic deletion and site-directed mutagenesis of this region identified four promoter regions*,* one driving *cts1L* transcription and three directing *cts1R* expression (P1, P2 and P3). Additional promoters were identified within the *cts1R* cluster. All *cts1* promoters were differentially repressed by H-NS. Moreover, expression of the response regulator CpxR from a low-copy plasmid enhanced promoter activity in the Δ*hns* background. Electrophoretic mobility shift assays showed that H-NS binds the *cts1* regulatory region directly, whereas phosphorylated CpxR bound the internal *cts1R* promoters but not the intergenic P1–P3 regions, suggesting that CpxR activates the latter indirectly. Together, these findings demonstrate that *cts1* expression is controlled by a complex hierarchical regulatory network in which H-NS acts as the primary repressor and CpxR contributes to activation only once H-NS-mediated repression is relieved, in a context-dependent manner. This multifactorial regulation likely involves additional, yet-unidentified factors that fine-tune *cts1* expression in response to environmental cues.

## Graphical Abstract

The CTS1 type VI secretion system of *Citrobacter rodentium* is organized into the divergent clusters: *cts1L* and *cts1R*. Multiple promoters coordinate *cts1* expression: four are located in the divergent regulatory region, and four additional promoters reside within *cts1R*. *cts1* is silenced by H-NS and activated by CpxR when repression is relieved.

**Impact Statement**

The type VI secretion system (T6SS) plays a central role in bacterial competition, niche establishment and survival within complex environments. Understanding how T6SS expression is regulated is essential for elucidating its biological functions. This study identifies key regulatory components controlling the CTS1 T6SS in *Citrobacter rodentium,* which is required to outcompete the mouse microbiota and successfully colonize the host. Our findings provide a framework to better understand how this pathogen modulates T6SS expression, revealing a complex hierarchical mechanism in which H-NS silences multiple *cts1* promoters and CpxR contributes to their activation upon derepression.

## Introduction

The type VI secretion system (T6SS) is a proteinaceous nanomachine that plays a central role in bacterial interactions and survival. It is widely distributed in Gram-negative bacteria, including pathogens, commensals, symbionts and free-living species that inhabit diverse ecological niches [1]. This system enables bacteria to deliver effector proteins into the target cell or the extracellular milieu [2]. The T6SS plays key roles in bacterial ecology and pathogenesis by mediating interbacterial competition, infection of eukaryotic cells and nutrient acquisition [3].

Assembly of the T6SS requires at least 14 conserved core components, designated TssA-M (T6SS), which assemble into a contractile apparatus structurally analogous to an inverted bacteriophage tail [4]. These proteins are typically encoded within a primary genomic cluster, which may also contain genes encoding Tag (T6SS-associated) proteins, as well as genes encoding effectors and their cognate immunity proteins. Up to six distinct T6SS clusters can coexist within a single genome [5], each differentially regulated in response to specific environmental or physiological signals [6–8].

Although the genetic organization and function of the T6SS have been extensively studied, its regulation is complex and often context-dependent and still not fully understood. In many organisms, T6SS gene clusters are tightly regulated and frequently remain silent under standard laboratory conditions, suggesting that their expression is restricted to specific environmental or host-associated niches [9–12].

T6SS expression is controlled by multiple regulatory mechanisms, including quorum sensing, TCSs, alternative sigma factors, enhancer-binding proteins, nucleoid-associated protein (NAPs) and a myriad of transcriptional regulators from diverse families (e.g. Fur, LysR, OmpR, AraC and others) [13, 14]. Some T6SS clusters also encode specific regulators, such as VP139 and VP1407, in *Vibrio parahaemolyticus* [15] and TagK in *Xanthomonas citri* [16].

Environmental factors, including temperature, pH, osmolarity, growth phase, nutrient availability, surface contact and metal ion concentration, further influence T6SS expression [14, 17]. Consequently, activation of certain T6SSs under standard laboratory conditions can be difficult to achieve [9, 18–20].

*C. rodentium* is a mouse-specific pathogen that causes transmissible murine colonic hyperplasia [21, 22]. It has been widely used as a model organism to study attaching and effacing pathogenesis [23], the development of human intestinal disorders, such as Crohn’s disease and ulcerative colitis [24] and the role of the intestinal microbiota in colonization resistance [25].

This pathogen encodes two T6SSs, designated *C. rodentium* type VI secretion system clusters 1 (*cts1*) and 2 (*cts2*) [26]. Both *cts1* and *cts2* are organized into two divergent gene clusters that encode the core structural and accessory components of their respective systems. Because *cts1* is not expressed under standard laboratory growth conditions, Gueguen and Cascales [9] developed a strategy to study CTS1 function by replacing its divergent regulatory region with IPTG- and arabinose-inducible promoters. They demonstrated that Hcp is secreted and that CTS1 contributes to interbacterial competition. More recently, *C. rodentium* was shown to use CTS1 to outcompete specific members of the mouse gut microbiota via secretion of the VgrG-1 effector, thereby facilitating gut colonization of the host intestine [20].

Although CTS1 functionality has been demonstrated, the regulatory mechanisms controlling its transcriptional expression remain poorly understood. In this study, we show that *cts1* expression depends on four promoters located within the divergent regulatory region – one driving *cts1L* and three driving *cts1R* (P1, P2 and P3) – as well as additional promoters within the *cts1R* cluster. These promoters are controlled by a complex hierarchical mechanism in which the global repressor H-NS silences the locus and the two-component response regulator CpxR contributes to activation upon derepression, acting directly at the internal promoters and indirectly at the divergent regulatory region.

## Methods

### Bacterial strains, plasmids and growth conditions

Bacterial strains and plasmids used in this study are listed in Table 1. Cultures were routinely grown in Luria-Bertani (LB) broth at 37 °C with shaking at 200 r.p.m. When indicated, bacteria were cultured in Dulbecco’s Modified Eagle’s Medium (DMEM; Gibco-BRL Life Technologies, Waltham, MA, USA) or in Minimal Medium N (MM-N; KCl 5 mM, (NH_4_)_2_SO_4_ 7.5 mM, K_2_SO_4_ 0.5 mM, KH_2_PO_2_ 1 mM, MgCl_2_ 200 µM, casamino acids 0.1 g l^−1^ and 0.48% [w/v] glucose) at 37 °C under static conditions in a 5% CO_2_ atmosphere (CO_2_ incubator). When required, antibiotics were added at the following concentrations: kanamycin (30 µg ml^−1^), ampicillin (200 µg ml^−1^) and tetracycline (15 µg ml^−1^).

**Table 1.** Strains and plasmids used in this study

Strain or plasmid	Genotype or description	Resistance	Reference/ Source
**Strain**			
*C. rodentium*			
DBS100	*C. rodentium* WT	No	[87]
JPCR13	DBS100 ∆*hns*::FRT	No	[36]
∆*cicR*	DBS100 ∆*hns2*::Km	Km	Isidro-Coxca, unpublished
JPCR3	DBS100 ∆*hns3*::Km	Km	[36]
JPCR4	DBS100 ∆*stpA*::Km	Km	[36]
JPCR5	DBS100 ∆*ler*::Km	Km	[36]
*Escherichia coli*			
DH10β	F– *mcrA* Δ(*mrr-hsdRMS-mcrBC*) φ80*lacZ*ΔM15 Δ*lacX74 recA1 endA1 araD139* Δ (*ara-leu*)7697 *galU galK* λ– *rpsL*(Str^R^) *nupG*	Str	[88]
BL21/pLys	Strain for expression of recombinant proteins	Cm	Invitrogen
**Plasmid**			
pKK232-8	pBR322 derivative containing a promoterless cat reporter gene	Ap	Pharmacia Biotec
pKK-cts1L-cat*	*cts1L* transcriptional fusion from nt −420 to +500	Ap	This study
pKK-cts1L_1-cat*	*cts1L* transcriptional fusion from nt −209 to +500	Ap	This study
pKK-cts1L_2-cat*	*cts1L* transcriptional fusion from nt −298 to +500	Ap	This study
pKK-cts1L_3-cat*	*cts1L* transcriptional fusion from nt −209 to +148	Ap	This study
pKK-cts1L_4-cat*	*cts1L* transcriptional fusion from nt −298 to +148	Ap	This study
pKK-cts1L_5-cat*	*cts1L* transcriptional fusion from nt −420 to +148	Ap	This study
pKK-cts1L_6-cat*	*cts1L* transcriptional fusion from nt −298 to −155	Ap	This study
pKK-cts1L_8-cat*	*cts1L* transcriptional fusion from nt −112 to +500	Ap	This study
pKK-cts1R-cat*	*cts1R* transcriptional fusion from nt −790 to +130	Ap	This study
pKK-cts1R_3-cat*	*cts1R* transcriptional fusion from nt −424 to +130	Ap	This study
pKK-cts1R_4-cat*	*cts1R* transcriptional fusion from nt −290 to +130	Ap	This study
pKK-cts1R_5-cat*	*cts1R* transcriptional fusion from nt −192 to +130	Ap	This study
pKK-cts1R_6-cat*	*cts1R* transcriptional fusion from nt −424 to +16	Ap	This study
pKK-cts1R_7-cat*	*cts1R* transcriptional fusion from nt −290 to +16	Ap	This study
pKK-cts1R_8-cat*	*cts1R* transcriptional fusion from nt −192 to +16	Ap	This study
pKK-cts1R_9-cat*	*cts1R* transcriptional fusion from nt −290 to −164	Ap	This study
pKK-cts1R_10-cat*	*cts1R* transcriptional fusion from nt −424 to −164	Ap	This study
pKK-cts1R_14-cat*	*cts1R* transcriptional fusion from nt −103 to +16	Ap	This study
pKK-cts1R_15-cat*	*cts1R* transcriptional fusion from nt −103 to +130	Ap	This study
pKK-cts1R_17-cat*	*cts1R* transcriptional fusion from nt −4 to +130	Ap	This study
pKK-cts1R_18-cat*	*cts1R* transcriptional fusion from nt −192 to −78	Ap	This study
pKK-*cts1L_mutP1-cat*	pKK-*cts1L*-cat derivative containing a modified −10 sequence of promoter 1 (TTTAAT to GAGCTC)	Ap	This study
pKK-*cts1R3_mutP1*_*B*_*-cat*	pKK-*cts1R3*-cat derivative with a modified −10 promoter 1 sequence (TAGAAT to GAGCTC)	Ap	This study
pKK-*cts1R3_mutP1*_*A*_*-cat*	pKK-*cts1R3*-cat derivative containing a modified −10 sequence of promoter 1 (TTAAAT to GAGCTC)	Ap	This study
pKK-*cts1R3_mutP2*_*B*_*-cat*	pKK-*cts1R3*-cat derivative containing a modified −10 sequence of promoter 2 (TACGCT to GAGCTC)	Ap	This study
pKK-*cts1R3_mutP2*_*A*_*-cat*	pKK-*cts1R3*-cat derivative containing a modified −10 sequence of promoter 2 (TAAATT to GAGCTC)	Ap	This study
pKK-*cts1R3_mutP3-cat*	pKK-*cts1R3*-cat derivative containing a modified −10 sequence of promoter 3 (TTTAGT to GAGCTC)	Ap	This study
pKK-*cts1R10_mutP1*_*B*_*-cat*	pKK-*cts1R10*-cat derivative containing a modified −10 sequence of promoter 1 (TAGAAT to GAGCTC)	Ap	This study
pKK-*cts1R10_mutP1*_*A*_*-cat*	pKK-*cts1R10*-cat derivative containing a modified −10 sequence of promoter 1 (TTAAAT to GAGCTC)	Ap	This study
pKK-*cts1R17_mutP2*_*B*_*-cat*	pKK-*cts1R17*-cat derivative containing a modified −10 sequence of promoter 2 (TACGCT to GAGCTC)	Ap	This study
pKK-*cts1R17_mutP2*_*A*_*-cat*	pKK-*cts1R17*-cat derivative containing a modified −10 sequence of promoter 2 (TAAATT to GAGCTC)	Ap	This study
pKK-*cts1R_18mutP3-cat*	pKK-*cts1R18*-cat derivative containing a modified −10 sequence of promoter 3 (TTTAGT to GAGCTC)		
pKK-*tsfA-cat*	*tsfA* transcriptional fusion	Ap	This study
pKK-*tssB-cat*	*tssB* transcriptional fusion	Ap	This study
pKK-*hcp-cat*	*hcp* transcriptional fusion	Ap	This study
pKK-*tssJ-cat*	*tssJ* transcriptional fusion	Ap	This study
pKK-*PAAR-cat*	*PAAR* transcriptional fusion	Ap	This study
pBAD-H-NS-FH	pBADMycHis derivative carrying H-NS-FH from an ara promoter	Ap	[89]
pQE32-CpxR	pQE32 derivative carrying the *E. coli cpxR* gene fused to His_6_	Ap	Oropeza-Navarro unpublished
pREP4	p15A derivative for regulating expression from pQE vectors	Km	Qiagen
pMPM-T3	p15A derivative low-copy-number cloning vector	Tc	[90]
pT3-CpxR	pMPM-T3 derivative carrying the *C. rodentium cpxR* gene under the control of the plasmid’s P*lac* promoter	Tc	This study

* pKK232-8 derivatives carrying *cts1L–cat* or *cts1R–cat* transcriptional fusions. Fragment coordinates are given relative to the *tssF* or *tssH* start codon.

### Construction of plasmids

Cloning steps were performed in *Escherichia coli* DH10β, and constructs were propagated in this strain for sequencing before introduction into *C. rodentium*. To generate *cat* transcriptional fusions, fragments of the *cts1* regulatory region were amplified by PCR with Phusion High-Fidelity DNA Polymerase (Thermo Fisher Scientific, Waltham, MA, USA) using specific oligonucleotides (Table 2) and *C. rodentium* genomic DNA as the template. PCR products were purified using the Biomiga DNA Gel PCR Purification Kit (Biomiga Inc., San Diego, CA, USA), digested with FastDigest BamHI and HindIII (Thermo Fisher Scientific, Waltham, MA, USA) and ligated into plasmid pKK232-8 (Table 1), digested with the same enzymes, using T4 DNA ligase (Thermo Fisher Scientific).

**Table 2.** Oligonucleotides used in this study

Name	Sequence 5'-3**'**	Use
tssH-F CAT Fw1	GGTAAGCTTTTAAGGCGATGGAGTTCCAG	Generation of *cts1L*, *cts1L_1*, *cts1L_2* and *cts1L_8* Amplification of *cts1* DNA fragment for EMSA
tssH-F CAT Fw2	TCAAAAGCTTTACGTAAGGATCGG	Generation of *cts1L_3*, *cts1L_4* and *cts1L_5*
tssH-F CAT Fw3	GGAAGCTTTGATTTAATATTTTCCT	Generation of *cts1L_6*
tssH-F CAT Rv1	AACCGGATCCTTCAACGTAAGGATTACCGC	Generation of *cts1L* and *cts1L_5*
tssH-F CAT Rv2	AGACGGATCCCTTTTTAATTCGATG	Generation of *cts1L_2*, *cts1L_4* and *cts1L_6*
tssH-F CAT Rv3	GAGGGATCCTCTGGCTACTTC	Generation of *cts1L_1* and *cts1L_3*
tssH-F CAT Rv4	GAGGGGATCCCTAATTTCACCGGA	Generation of *cts1L_8*
tssF-H CAT Fw1	GGTGGATCCTTAAGGCGATGGAGTTCCAG	Generation of *cts1R*
tssF-H CAT Fw3	ATTGGATCCCGTCAATTTTCGCGACTTG	Generation of *cts1R_4, cts1R_7* and *cts1R_9*
tssF-H CAT Fw4	TTTGGATCCTCTCCGGTGAAATTAGGAATCC	Generation of *cts1R_5, cts1R_8* and *cts1R_18*
tssF-H CAT Fw5	TAAGGATCCGCAGTTTCTATCCCC	Generation of *cts1R_3, cts1R_6* and *cts1R_10*
tssF-H CAT Fw7	CGAAGGATCCAGAGGATGCCTCTGTTA	Generation of *cts1R_14* and *cts1R_15*
tssF-H CAT Fw9	AGGGATCCGTCTTGTTGTGGCGATTA	Generation of *cts1R_17*
tssF-H CAT Rv	AACCAAGCTTTTCAACGTAAGGATTACCGC	Generation of *cts1R*, *cts1R_3*, *cts1R_4*, *cts1R_5*, *cts1R_15* and *cts1R_17* Amplification of *cts1* DNA fragment for EMSA
tssF-H CAT Rv1	CACAAGCTTGACTCATCCCTTTTTAATTCG	Generation of *cts1R_6*, *cts1R_7*, *cts1R_8* and *cts1R_14*
tssF-H CAT Rv2	GAAAGCTTACTCAGAGGGGATTCCTAAT	Generation of *cts1R_9* and *cts1R_10*
tssF-H CAT Rv4	ACAGAAGCTTCCTCTGGCTACT	Generation of *cts1R_*18
cts1L-P1P-Fw	CGCAGAGCTCTCTAGAATCCGG	Generation of *l-P1*
cts1L-P1P-Rv	TAGAGAGCTCTGCGTTCTTCAC	Generation of *l-P1*
cts1R-P1P-Fw	GATTCGAGCTCTAAATGCGTTCTTCAC	Generation of *R3-P1_A_* and *R10-P1_A_*
cts1R-P1P-Rv	TTTAGAGCTCGAATCCGGCGCTAT	Generation of *R3-P1_B_* and *R10-P1_B_*
cts1R-P1-Fw	AGAAGAGCTCGCGTTCTTCACGAT	Generation of *R3-P1_A_* and *R10-P1_A_*
cts1R-P1-Rv	TTTAGAGCTCGAATCCGGCGCTAT	Generation of *R3-P1_A_* and *R10-P1_A_*
cts1R-P2P-Fw	CCACGAGCTCGTGTAAATTACGCG	Generation of *R3-P2_B_* and *R17-P2_B_*
cts1R-P2P-Rv	TACACGAGCTCGTGGCACTCTCCA	Generation of *R3-P2_B_* and *R17-P2_B_*
cts1R-P2-Fw	TGTGGAGCTCACGCGGTAATCCTTA	Generation of *R3-P2_A_* and *R17-P2_A_*
cts1R-P2-Rv	GCGTGAGCTCCACAGCGTAGTGGCA	Generation of *R3-P2_A_* and *R17-P2_A_*
cts1R-P3-Fw	TATGGAGCTCATTAACGCGAAGTAGC	Generation of *R3-P3* and *R18-P3*
cts1R-P23Rv	GTAATGAGCTCCATAGTTGTCGATAAGG	Generation of *R3-P3* and *R18-P3*
27801 CAT Fw	AGAAAAGGATCCTACCAAAGATAGGCAGTGCAC	Generation of *tsfA* transcriptional fusion Amplification of *tsfA* DNA fragment for EMSA
27802 CAT Rv	AGTCTAAAGCTTACGTTGATACGCTCGCCATC	Generation of *tsfA* transcriptional fusion Amplification of *tsfA* DNA fragment for EMSA
tssB CAT Fw	CGGCGGATCCATTAAGCCATAGATTT	Generation of *tssB* transcriptional fusion Amplification of the *tssB* DNA fragment for EMSA
tssB CAT Rv	CGCAAGCTTGCCCATCACGAACGGCAATT	Generation of *tssB* transcriptional fusion Amplification of *tssB* DNA fragment for EMSA
hcp CAT Fw	AAGGGGATCCGGGTTATTACCAGGCGAA	Generation of *hcp* transcriptional fusion Amplification of *hcp* DNA fragment for EMSA
hcp CAT Rv	CTTCAAGCTTTGCTTTACCTGCGCC	Generation of *hcp* transcriptional fusion Amplification of *hcp* DNA fragment for EMSA
tssJ CAT Fw	GGCAGGATCCAACGAAGACGAAACA	Generation of *tssJ* transcriptional fusion
tssJ CAT Rv	TTTAAAGCTTCGCCATCGCTCGcc	Generation of *tssJ* transcriptional fusion
PAAR CAT Fw	GACGGATCCCCCAACAGAGACACCAA	Generation of *PAAR* transcriptional fusion Amplification of *PAAR* DNA fragment for EMSA
PAAR CAT Rv	TCCAAAGCTTCCGCGACTTACC	Generation of *PAAR* transcriptional fusion Amplification of *PAAR* DNA fragment for EMSA
tssF-G Fw	AATCGCGCGCATTGTGCTAA	Reverse transcription PCR (RT-PCR)
tssF-G Rv	TTCTCCGGCATCAGTCCGTAT	RT-PCR
tssG-A Fw	ATGATGAATGCGGGGTCGTA	RT-PCR
tssG-A Rv	GGAGCGTAATGCTTGTGATG	RT-PCR
tssA-pexp Fw	CGCATTCGCGTTGATACGAA	RT-PCR
tssA-pexp Rv	CGCTTATTCAGGTGCTGAAG	RT-PCR
pexp-ph Fw	ACGGAGAGATGCAGATTCTC	RT-PCR
pexp-ph Rv	AAACCAGCATTACCAGCCAG	RT-PCR
cts1O-cts1W Fw	TCCTCATCCATATAGCGCAG	RT-PCR
cts1O-cts1W Rv	CATTAAAGAGTGGCTGGTGG	RT-PCR
gcfA-Fw	GAGTTTGGATCCCGCTATGGCTATTGACGGTAC	RT-PCR
gcfA-Rv	AACCCAAAGCTTGAACAACCAACGAGGAAAGAAG	RT-PCR
ROD_27771 FLAG Fw	TGACAAACCAGTTCAAGGGGGA	Amplification of *ctrl (-*) (233 bp fragment) DNA fragment for EMSA
fl7A_pBAD Fw	CACCCATGGTCACGCAGGGTACCGTAAC	Amplification of ctrl (-) (488 bp fragment) DNA fragment for EMSA
fl7A_pBAD Rv	CGGAAGCTTCGGATACGCGATGGTATAAGC	Amplification of *ctrl (-*) (233 and 488 bp fragments) DNA fragments for EMSA
cpxP-cat Fw	AAAGGGATCCCAACTCTCGGTCATCATC	Amplification of *cpxP* DNA fragment for EMSA
cpxP-cat Rv	TGCAAGCTTTGAGGCCATGACGGCA	Amplification of *cpxP* DNA fragment for EMSA
CpxRA-HindIII Fw	TAAAGCTTTGGATTAGCGGCGC	Generation of pT3-CpxR
CpxR-BamHI Rv	CAGGATCCACACCAGCATCAGC	Generation of pT3-CpxR
tssH-prox	GCACTCTCCACGCTTTTAAAC	5′ rapid amplification of cDNA end (5′ RACE)
tssH-dist	CTGAGCAAAATCACGTTCGAG	5′ RACE

Underlined letters indicate the corresponding restriction sites.

Promoter-region mutations were generated by inverse PCR with Phusion High-Fidelity DNA Polymerase, using the corresponding *cat* transcriptional fusion constructs as templates and overlapping primers (Table 2) designed to replace the targeted promoter sequences with a SacI restriction site. PCR amplicons were purified using the Gel DNA Recovery Kit (Zymoclean, Irvine, CA, USA), digested with FastDigest SacI (Thermo Fisher Scientific) and re-ligated using T4 DNA ligase (Thermo Fisher Scientific).

For construction of the pT3-CpxR plasmid (Table 1), the *cpxR* coding sequence was amplified by PCR, purified, digested with BamHI and HindIII and ligated into the pMPM-T3 vector (Table 1) using T4 DNA ligase (Thermo Fisher Scientific).

The resulting pKK232-8 and pMPM-T3-derived plasmids (Table 1) were introduced into *C. rodentium* strains by electroporation. All constructs were verified by DNA sequencing.

### Chloramphenicol acetyltransferase assays

Chloramphenicol acetyltransferase (CAT) activity was measured as previously described [27]. Briefly, bacterial cultures were grown in 5 ml of LB, DMEM or MM-N for 12 h under static conditions in a 5% CO_2_ atmosphere. A total of 1.5 ml of cells was harvested by centrifugation, washed and resuspended in 300 µl of TDTT buffer (50 mM Tris-HCl, pH 7.8 and 30 µM dl-Dithiothreitol). Total soluble cell extracts were prepared by sonication using a Soniprep 150 (MSE Ltd, UK) and clarified by centrifugation. Supernatants were transferred to fresh tubes.

For the CAT assay, 5 µL of each extract was mixed with 200 µL of reaction solution (1 mM DTNB (5,5′-dithiobis-(2-nitrobenzoic acid)), 0.1 M Tris-HCl pH 7.8, 0.1 mM acetyl-CoA and 0.1 mM chloramphenicol) in a 96-well plate. Absorbance at 410 nm was recorded every 5 s for 5 min using a CERES 900C microplate reader operated with the KC3 software in kinetic mode (Bio-Tek Instruments, Winooski, VT, USA).

Protein concentrations were determined by mixing 10 µl of each extract (in duplicate) with 200 µl of the colorimetric reagent prepared by combining 25 ml of solution A with 500 µl of solution B from the BCA Protein Assay Reagent Kit (Pierce, Rockford, IL, USA) in a 96-well plate. The mixtures were incubated at 37 °C for 30 min and absorbance was measured at 562 nm using the same microplate reader.

All assays were performed in at least three independent experiments, each conducted in duplicate.

### Expression and purification of H-NS and CpxR proteins

*E. coli* BL21/pLysS strains carrying either pBAD-H-NS-FH or pQE32-CpxR (Table 1) were grown in 100 ml of LB medium at 37 °C with shaking. When cultures reached an optical density at 600 nm of 0.6, protein expression was induced by adding 1 mM isopropyl-*β*-d-thiogalactopyranoside, followed by incubation for an additional 4 h at 30 °C. Cells were harvested by centrifugation at 5,000 r.p.m. for 10 min at 4 °C, washed once with 50 ml of cold binding buffer (5 mM imidazole, 20 mM Tris-HCl pH 8.0, 500 mM NaCl) and resuspended in 30 ml of the same buffer.

The bacterial suspension was lysed by three passes through a French press at 1,200 psi. Cell debris was removed by centrifugation at 7,000 r.p.m. for 40 min at 4 °C and the resulting supernatant was applied to a propylene column containing 2.5 ml of Ni-NTA agarose resin (Qiagen, Hilden, Germany) pre-equilibrated with binding buffer. The column was washed sequentially with ten column volumes of binding buffer and ten column volumes of washing buffer (20 mM imidazole, 20 mM Tris-HCl pH 8.0, 500 mM NaCl). Bound proteins were eluted with 10 ml of elution buffer (250 mM imidazole, 20 mM Tris-HCl pH 8.0, 500 mM NaCl).

Eluted fractions were analysed by SDS/PAGE, and protein concentrations were determined using the Bradford assay.

### Electrophoretic mobility shift assays

Electrophoretic mobility shift assays (EMSAs) were performed using purified H-NS and CpxR proteins and PCR-amplified fragments spanning different segments of the *cts1* regulatory region. Two DNA fragments of 233 and 488 bp corresponding to the *ROD_27801* structural gene were used as negative controls. CpxR was phosphorylated with 50 mM acetyl phosphate (Sigma-Aldrich, St. Louis, MO, USA) in elution buffer (2 M imidazole, 5 M NaCl, 2 M Tris-HCl pH 8) supplemented with 10 mM MgCl_2_ for 1 h at 30 °C. The oligonucleotides used for PCR amplification are listed in Table 2.

For H-NS binding assays, 50 ng of each DNA fragment was incubated with increasing concentrations of purified H-NS in buffer containing 400 mM HEPES, 80 nM MgCl_2_, 500 nM KCl, 10 µM DTT, 0.5% NP40 nonidet and 1 mg ml^−1^ BSA. For phosphorylated CpxR (CpxR-P) binding, DNA fragments were incubated with increasing concentrations of CpxR-P in buffer containing 20 mM Tris-HCl pH 7.5, 50 mM KCl, 1 mM dithiothreitol and 5% glycerol [28]. Reaction mixtures were incubated for 20 min at 30 °C and resolved by electrophoresis on 5.5% polyacrylamide gels in 0.5× Tris-borate-EDTA buffer at 80 V for 2.5–4.5 h at 4 °C. Gels were stained with ethidium bromide and visualized using a Molecular Imager Gel Doc XR System (Bio-Rad, Hercules, CA, USA).

### Reverse transcription PCR analysis of *cts1* transcripts

*C. rodentium* Δ*hns* was grown in MM-N for 12 h at 37 °C in a 5% CO_2_ atmosphere. A total of 20 ml culture was harvested by centrifugation and total RNA was purified using RNeasy Mini Kit (Qiagen, Hilden, Germany). RNA samples were treated with DNase I (Fermentas, Pittsburgh, PA, USA) for 30 min at 37 °C to remove residual DNA, followed by heat inactivation at 65 °C for 10 min. cDNA synthesis was performed in a 20 µl reaction containing 1 µg of total RNA, the *cts1O-cts1W Rv* primer and 200 U of Maxima H Minus Reverse Transcriptase (Thermo Fisher, Waltham, MA, USA). PCR amplification was carried out with 1 µL of the cDNA reaction as a template and specific oligonucleotides (Table 2) to amplify different regions of *cts1*. PCR products were resolved by electrophoresis on 1% agarose gels and visualized with ethidium bromide under UV illumination.

### 5′ rapid amplification of cDNA end

5′ rapid amplification of cDNA end (5′ RACE) was performed as previously described [29]. Briefly, total RNA was purified from * C. rodentium* Δ*hns* grown in MM-N for 12 h at 37 °C in 5% CO_2_ atmosphere using the RNeasy Mini Kit (Qiagen, Hilden, Germany) and treated with DNase I (Fermentas, Pittsburgh, PA, USA) to remove residual DNA. cDNA was synthesized from 1 µg of total RNA using random hexamer primers in a 20 µl reaction containing 200 U of SuperScript III Reverse Transcriptase (Invitrogen, Carlsbad, CA) according to the manufacturer’s instructions. The resulting cDNA was purified and enzymatically labelled at its 3′ end with homopolymeric poly(A) tail using 20 U of terminal deoxynucleotidyl transferase (Fermentas, St. Leon-Rot, Germany). PCR amplification was performed using an oligo(dT) primer and specific *cts1* primers (Table 2). PCR products were purified and sequenced to determine the transcription start site (TSS). The obtained sequence fragments were used solely to map the TSS and do not represent newly characterized sequence data.

### Statistical analysis

Data were analysed using the two-tailed Mann–Whitney U test in GraphPad Prism 6 GraphPad Software, San Diego, CA, USA.

## Results

### The expression of the *cts1* cluster is differentially regulated by the growth medium in the absence of H-NS

**Fig. 1.**
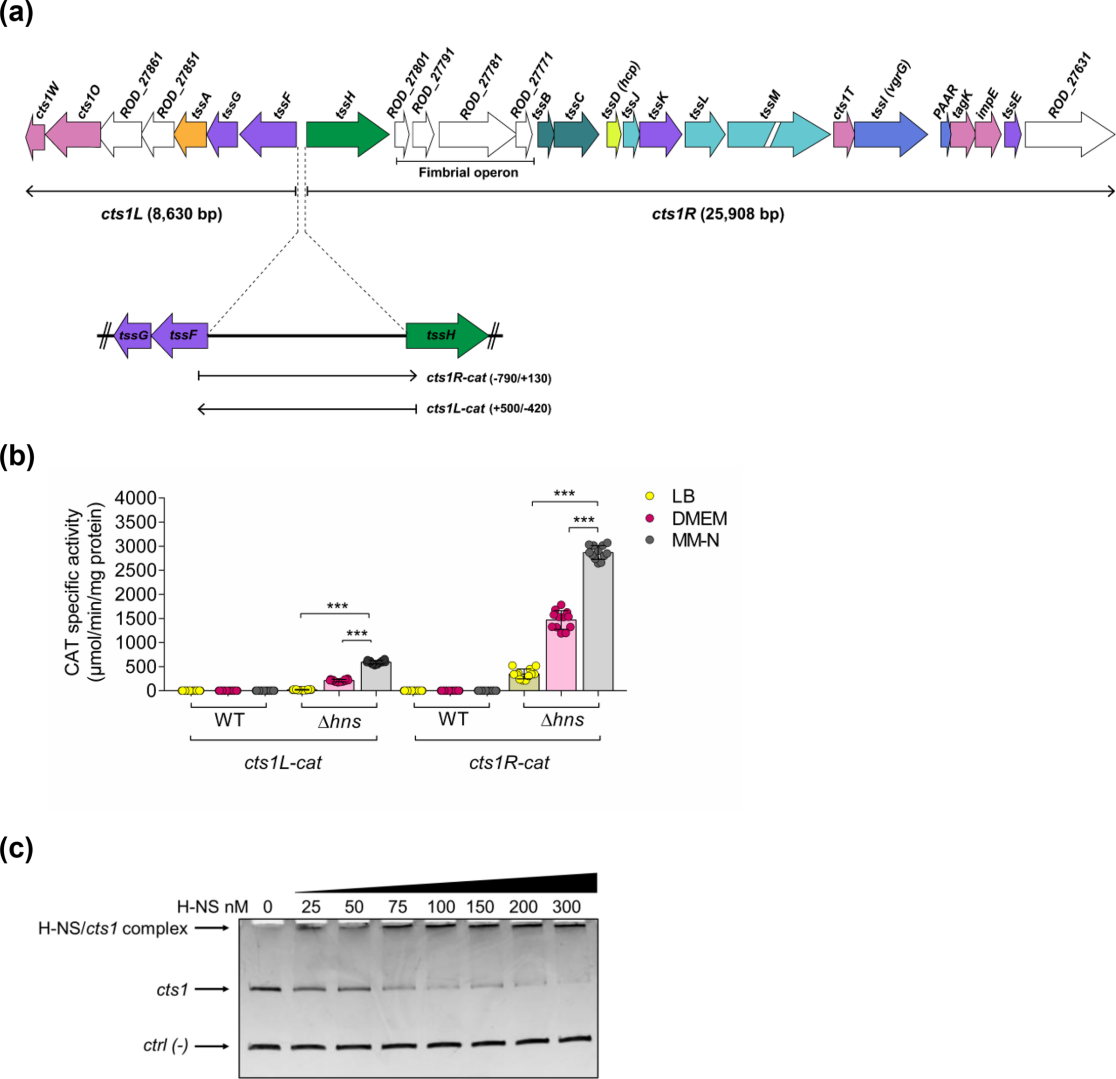
The *cts1* cluster is repressed by H-NS and differentially regulated by the growth medium. **(a**) Schematic representation of the *cts1* locus. The *cts1L* cluster comprises seven genes, while *cts1R* contains nineteen. Genes encoding core components are colour-coded as follows: purple, base plate; dark green, contractile sheath; light blue, membrane complex; dark blue, spike; orange, cap; yellow, internal tube; green, recycling; pink, other T6SS-associated proteins; and white, other non-T6SS-associated proteins. A magnified view of the divergent regulatory region between *cts1L* and *cts1R* is shown, indicating the *cts1L-cat* and *cts1R-cat* transcriptional fusions. Coordinates between parentheses refer to the *tssF* (GTG) and *tssH* (ATG) start codon, respectively. (**b**) Expression of the *cts1L-cat* and *cts1R-cat* transcriptional fusions in *C. rodentium* WT and ∆*hns* strains. CAT-specific activity was measured from cultures grown for 12 h at 37 °C in LB, DMEM and MM-N under a 5% CO_2_ atmosphere. Bars represent the mean±sd from six independent assays, each performed in duplicate. Statistical significance was determined by a two-tailed Mann–Whitney U test: ns *P*>0.05, *P≤0.05, **P≤0.01, ***P≤0.001, ****P≤0.0001. (**c**) EMSA showing binding of purified H-NS-FLAG-His to the *cts1L-cts1R* regulatory region (554 bp: positions −424 to +130 relative to *tssH* ATG). DNA fragments (50 ng) were incubated with increasing concentrations (0–300 nM) of H-NS. An internal fragment of the *ROD_27801* coding region (233 bp) was used as a negative control, *ctrl (-*). This is a representative result from four independent experiments that yielded similar results.

The *cts1* cluster is organized into two divergent gene sets, herein referred to as *cts1L* and *cts1R* (Fig. 1a). The *cts1L* region contains *tssF*, *tssG* and *tssA,* along with four genes of unknown function (*ROD_27851*, *ROD_27861*, *cts1O* and *cts1W*). In contrast, *cts1R* comprises most genes encoding T6SS components (*tssH*, *tssB*, *tssC*, *hcp*, *tssJ*, *tssK*, *tssL*, *tssM*, *vgrG, PAAR* and *tssE*), the accessory gene *tagK*, a putative operon (*ROD_27771–27801*) predicted to encode a chaperone-usher pilus, a putative autotransporter gene (*ROD_27631*) and two additional genes of unknown function (*cts1T* and *impE*).

Previous studies showed that *cts1* genes are not expressed under standard laboratory growth conditions [9, 20]. The cryptic nature of this gene cluster is, in part, attributed to the repression by the global regulator H-NS [20], a NAP known to negatively regulate numerous genes in pathogenic bacteria [30]. Consistent with this, the *cts1* locus is AT-rich (the *cts1L–cts1R* intergenic region has an A-T content of 60%, above the genome average of ∼46%), a feature characteristic of horizontally acquired regions that are targeted and silenced by H-NS and that frequently contain promoter-like sequences giving rise to pervasive transcription [31]. To identify growth conditions that could enable *cts1* expression, we constructed transcriptional fusions of the *cts1* regulatory region in both orientations to the promoterless *cat* reporter gene in plasmid pKK232-8 (Table 1 and Fig. 1a). The resulting plasmids pKK-*cts1L-cat* and pKK-*cts1R-cat* were introduced into WT *C. rodentium* and its isogenic Δ*hns* mutant, which were then grown under various culture conditions (Table S1, available in the online Supplementary Material).

No expression was detected under any of the tested conditions in WT *C. rodentium*. However, in the absence of H-NS, different levels of expression were observed in LB, DMEM and MM-N, with the highest expression detected in MM-N at 37 °C under a 5% CO_2_ atmosphere (Fig. 1b). Based on this observation, we selected these culture conditions for subsequent experiments. These results confirmed that H-NS represses not only the expression of the *cts1R* cluster, as previously reported [20], but also that of *cts1L*. However, the promoter activity of the *cts1R-cat* fusion was ∼fourfold higher than that of *cts1L-cat*.

H-NS is a global repressor that can polymerize along DNA to form protein filaments [32]. In some cases, these nucleoprotein filaments act as a barrier that prevents RNA polymerase binding to DNA [33]. Alternatively, H-NS filaments can obstruct transcription elongation or mediate the formation of DNA loops that trap RNA polymerase [34, 35]. To determine whether H-NS directly represses *cts1* by binding to its divergent regulatory region, we performed EMSA. Increasing concentrations of purified H-NS-FLAG-His protein were incubated with 50 ng of a PCR-amplified fragment encompassing the *cts1* divergent regulatory region. A concentration-dependent mobility shift was observed (Fig. 1c). As a negative control, a fragment corresponding to the coding region of *ROD_27801* was included, which showed no interaction with H-NS, confirming the specificity of H-NS binding to the *cts1* regulatory region.

*C. rodentium* encodes four additional H-NS-like proteins: StpA, H-NS2, H-NS3 and Ler [26, 36]. H-NS and StpA have been reported to interact and form heterodimers that cooperatively repress gene expression [37]. To assess whether these H-NS paralogues are involved in *cts1* regulation, we introduced the *cts1L-cat* and *cts1R-cat* transcriptional fusions into the *C. rodentium* Δ*stpA*, Δ*hns2*, Δ*hns3* and Δ*ler* strains (Table 1). Both *cts1L* and *cts1R* expression were repressed by H-NS*,* although a slight increase in *cts1R* activity was observed in the absence of StpA (Fig. S1). These findings indicate that H-NS is the primary repressor of *cts1* expression under the conditions tested.

### Characterization of the *cts1R* divergent regulatory region

**Fig. 2.**
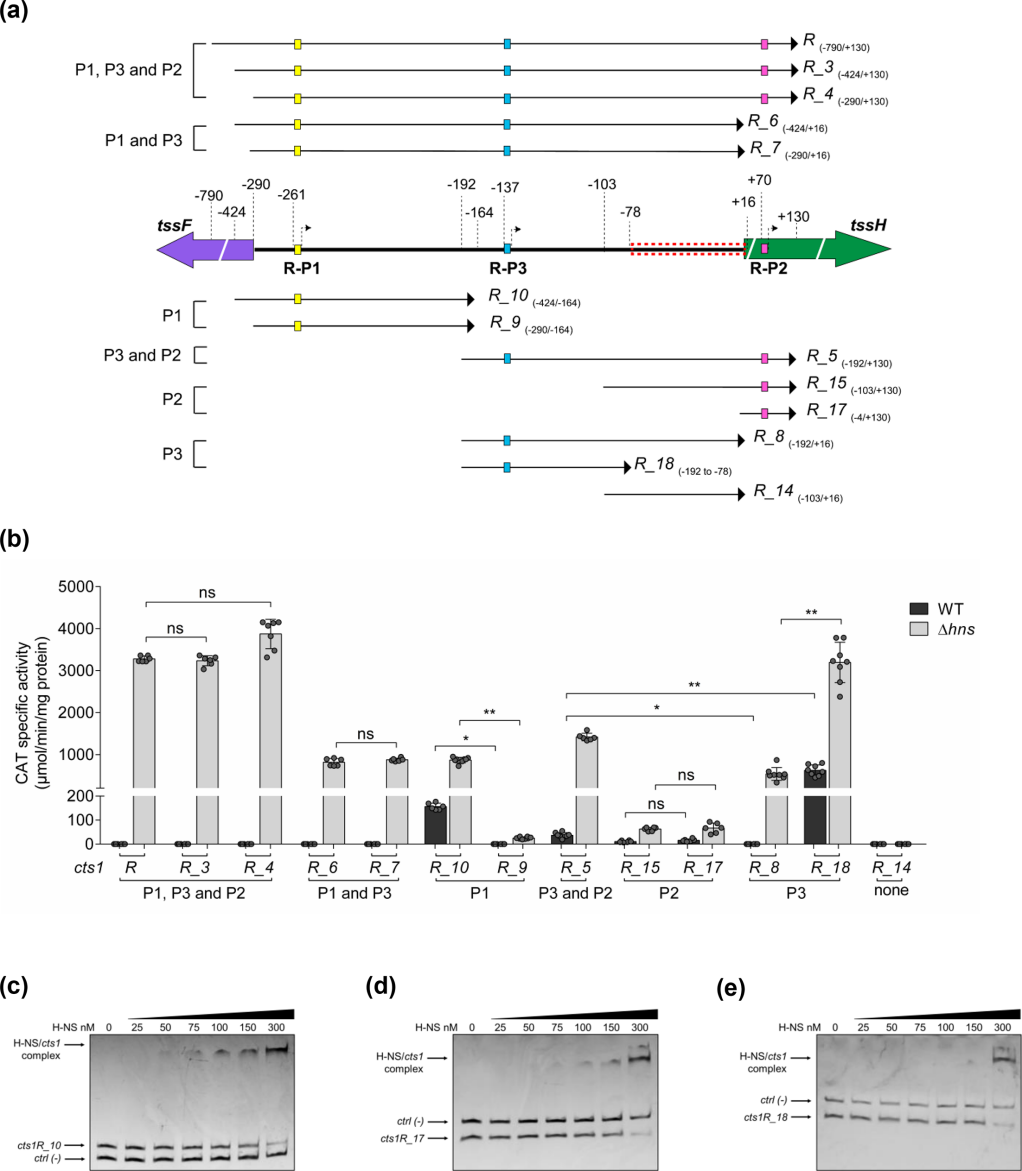
Characterization of the *cts1R* regulatory region.**(a**) Schematic representation of the *cts1R* regulatory region. Putative promoters are shown as yellow, pink and blue boxes; the approximate positions of the R-P1, R-P2 and R-P3 promoters are indicated; their −10 elements are analysed in detail in Fig. 4. Black arrows indicate the DNA fragments used to construct the *cts1R* transcriptional fusions: R, R_3-R_10, R_14-15 and R_17-18. The red dotted rectangle indicates the region associated with reduced expression. (**b**) Expression of the *cts1R* transcriptional fusions in *C. rodentium* WT and ∆*hns* mutant strains. CAT-specific activity was measured as described in Fig. 1. Bars represent the mean±sd from three independent assays performed in duplicate. Statistical significance was determined by a two-tailed Mann–Whitney U test: ns *P*>0.05, *P≤0.05, **P≤0.01, ***P≤0.001, ****P≤0.0001. (**c–e**) EMSA showing binding of purified H-NS-FLAG-His to the *cts1* regulatory region fragments *cts1R_10* (260 bp), *cts1R_17* (126 bp) and *cts1R_18* (114 bp). DNA fragments (50 ng) were incubated with increasing concentrations (0–300 nM) of H-NS. An internal fragment of *ROD_27801* coding region (233 bp) was used as a negative control, *ctrl (-*). This is a representative result from three independent experiments that yielded similar results.

To dissect the *cts1L-cts1R* divergent regulatory region, we constructed sixteen additional transcriptional fusions by deleting different portions of the sequence encompassed by the *cts1R-cat* fusion (Fig. 1a), which spans from positions −790 to +130 relative to the *tssH* ATG start codon; note that the +130 end lies within the *tssH* coding sequence, so promoter elements identified in this region may overlap the 5′ end of *tssH*. For clarity, only twelve of these constructs, which provided the most informative results, plus *cts1R-cat,* are shown in Fig. 2a. These fusions were designed to comprehensively cover the regulatory region and were evaluated in WT and Δ*hns C. rodentium* strains (Fig. 2b).

We found that *cts1R_10-cat, cts1R_17-cat* and *cts1R_18-cat* showed activity, even though they contain different segments of the *cts1L-cts1R* regulatory region, suggesting the presence of at least three functional promoters: R-P1, R-P2 and R-P3 (Fig. 2).

The *cts1R_10-cat* construct, containing only the putative R-P1, showed reduced activity compared with *cts1R-cat* in the ∆*hns* mutant, likely because of the absence of downstream positive regulatory elements. Interestingly, this fusion also showed low but detectable activity in the WT strain. In contrast, *cts1R_9-cat,* which also contains only R-P1, displayed almost no activity in the Δ*hns* mutant and none in the WT strain, indicating that the region between positions −424 and −290 is required for R-P1 functionality.

The *cts1R_18-cat* exhibited elevated expression levels in both WT and Δ*hns* strains. It displayed the highest activity in WT and expression levels comparable to *cts1R_3-cat* and *cts1R_4-cat,* the longest fusions containing all the putative promoters, in the Δ*hns* background. This promoter, R-P3, appears to be the strongest promoter under these conditions. In contrast, the *cts1R_15-cat* and *cts1R_17-cat* constructs*,* containing only the putative R-P2 promoter, exhibited lower activity, indicating that R-P2 is the weakest promoter under the tested conditions.

The *cts1R_14-cat* construct served as a control, as it showed no detectable activity, indicating the absence of additional promoters between positions −103 and +16.

The *cts1R_8-cat* construct, which also contains only R-P3, showed lower activity than *cts1R_18-cat*. Because these two fusions share the same promoter but differ in their downstream sequence, this difference could reflect a negative regulatory element between positions −78 and +16 or, alternatively, effects of the altered 5′ region on mRNA folding, stability or transcription elongation; we cannot formally distinguish these possibilities. This region appears distinct from H-NS-mediated repression, as *cts1R_18-cat* expression increased in the Δ*hns* mutant relative to WT.

The *cts1R_5-cat* construct, containing both R-P2 and R-P3, was more active than fusions containing only R-P2 but less active than *cts1R_18-cat*, consistent with the influence of the −78/+16 region described above. Similarly, fusions containing R-P1 and R-P3 (*cts1R_6-cat* and *cts1R_7-cat*) were more active than *cts1R_8-cat* (R-P3 only), reflecting the contribution of R-P1, but less active than *cts1R_18-cat*, again consistent with the effect of the −78/+16 region.

The *cts1R_3-cat* construct*,* which contains all three putative promoters, exhibited activity levels comparable to the full-length *cts1R-cat* and was identified as the minimal regulatory region required for *cts1R* expression. Therefore, *cts1R_3-cat* was used as a control in subsequent experiments.

Notably, we observed detectable expression from several constructs in the WT strain. Considering the ability of H-NS to form nucleoprotein complexes that adopt filamentous or bridged conformations on DNA [38], the reduced size of some DNA fragments may limit the formation of stable H-NS repression complexes [39]. Nevertheless, expression levels were consistently higher in the Δ*hns* strain, indicating that H-NS is still able to regulate these DNA fragments.

Remarkably, promoter activity is not simply additive across fusions, consistent with the influence of flanking sequences and/or the regulatory and RNA-level effects noted above. All promoters were repressed by H-NS, underscoring the need for a positive regulatory factor to counteract this repression. To explore whether H-NS could interact with multiple regions of the divergent regulatory sequence, we performed EMSA assays using DNA fragments encompassing the R-P1 (*cts1R_10-cat*), R-P2 (*cts1R_17-cat*) and R-P3 (*cts1R_18-cat*) promoter regions. H-NS bound all three fragments (Fig. 2c–e), consistent with the presence of more than one H-NS-binding region across the regulatory region. We note that these assays do not resolve the precise position of the individual H-NS sites relative to the promoters.

Together, our results suggest the existence of three promoter elements and identify a region that negatively affects *cts1R* expression, indicating the involvement of additional regulatory elements beyond H-NS.

### Characterization of the *cts1L* divergent regulatory region

**Fig. 3.**
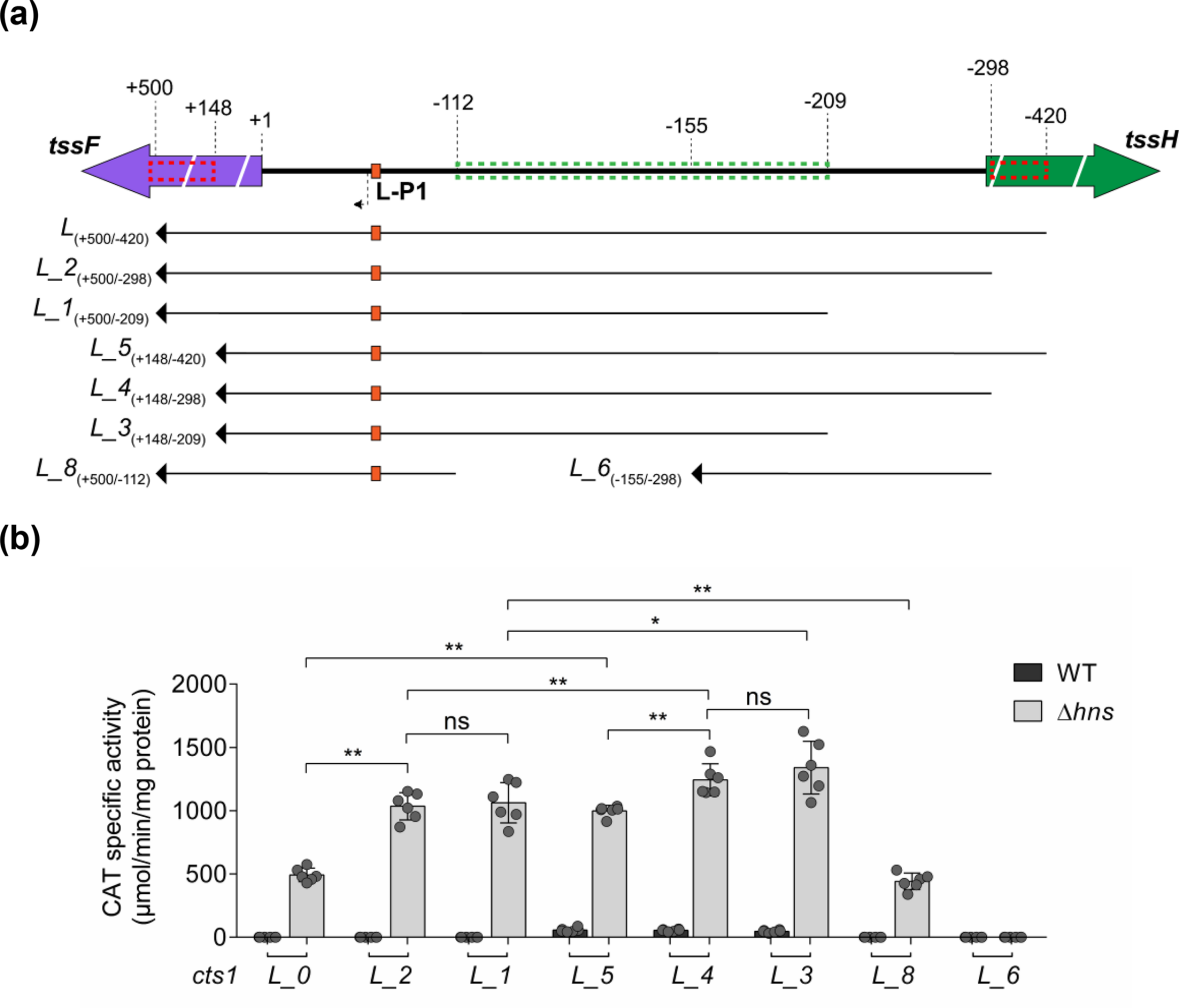
Analysis of the *cts1L* regulatory region. **(a**) Schematic representation of the *cts1L* regulatory region. The orange box and the downstream arrow denote the putative promoter and the TSS predicted in silico. Black arrows indicate the DNA fragments used to construct *cts1L* transcriptional fusions (L, L_1-6 and L_8). Green dotted rectangle, positive regulatory region (−209 to −112); red dotted rectangles, regions associated with reduced expression. (**b**) Expression of the *cts1L* transcriptional fusions in *C. rodentium* WT and ∆*hns* mutant strains. CAT-specific activity was measured as described in Fig. 1. Bars represent the mean±sd from three independent assays performed in duplicate. Statistical significance was determined by a two-tailed Mann–Whitney U test: ns *P*>0.05, *P≤0.05, **P≤0.01, ***P≤0.001, ****P≤0.0001.

Similarly to the previous section, we generated seven additional transcriptional fusions by deleting portions of the *cts1L-cat* region (Fig. 1a), which spans from −420 to +500 relative to the *tssF* GTG start codon (Fig. 3a). All fusions were evaluated in WT and Δ*hns C. rodentium* strains (Fig. 3b).

The *cts1L_2-cat* construct exhibited higher expression than *cts1L-cat,* suggesting the presence of a region that reduces expression between positions −420 and −298. *cts1L_1-cat* and *cts1L_2-cat* showed similar expression levels, indicating the absence of regulatory elements between positions −298 and −209. Conversely, *cts1L_5-cat* showed increased expression relative to *cts1L-cat*, defining a region between +148 and +500 that reduces expression. As for the *cts1R* fusions, because these constructs share the same promoter but differ in downstream sequence (*cts1L-cat* vs *cts1L_5-cat*), this difference could reflect a downstream regulatory element or, alternatively, effects on mRNA folding, stability or transcription elongation. *cts1L_4-cat* was more active than *cts1L_5-cat,* confirming the repressive role of the −420 and −298 region, while *cts1L_3-cat* and *cts1L_4-cat* showed similar activity, further supporting the lack of regulation between −298 and −209. *cts1L_3-cat* was also more active than *cts1L-cat,* consistent with the removal of the +148 to +500 repressive region.

Notably, *cts1L_8-cat* showed reduced expression compared to *cts1L_1-cat,* suggesting a positive regulatory region between −209 and −112. In contrast, *cts1L_6-cat* showed no detectable activity, likely due to the absence of a functional promoter.

In contrast to *cts1R,* this analysis identified only a single putative promoter shared by all tested fusions, except *cts1L_6-cat*, driving *cts1L* expression. However, we identified two regions involved in *cts1L* repression (+148 to +500 and −420 to −298 relative to *tssF* GTG) and a region associated with activation (−209 to −112 relative to *tssF* GTG), suggesting the likely presence of an unknown activator that modulates the *cts1L* expression.

### Functional analysis of the putative promoters

**Fig. 4.**
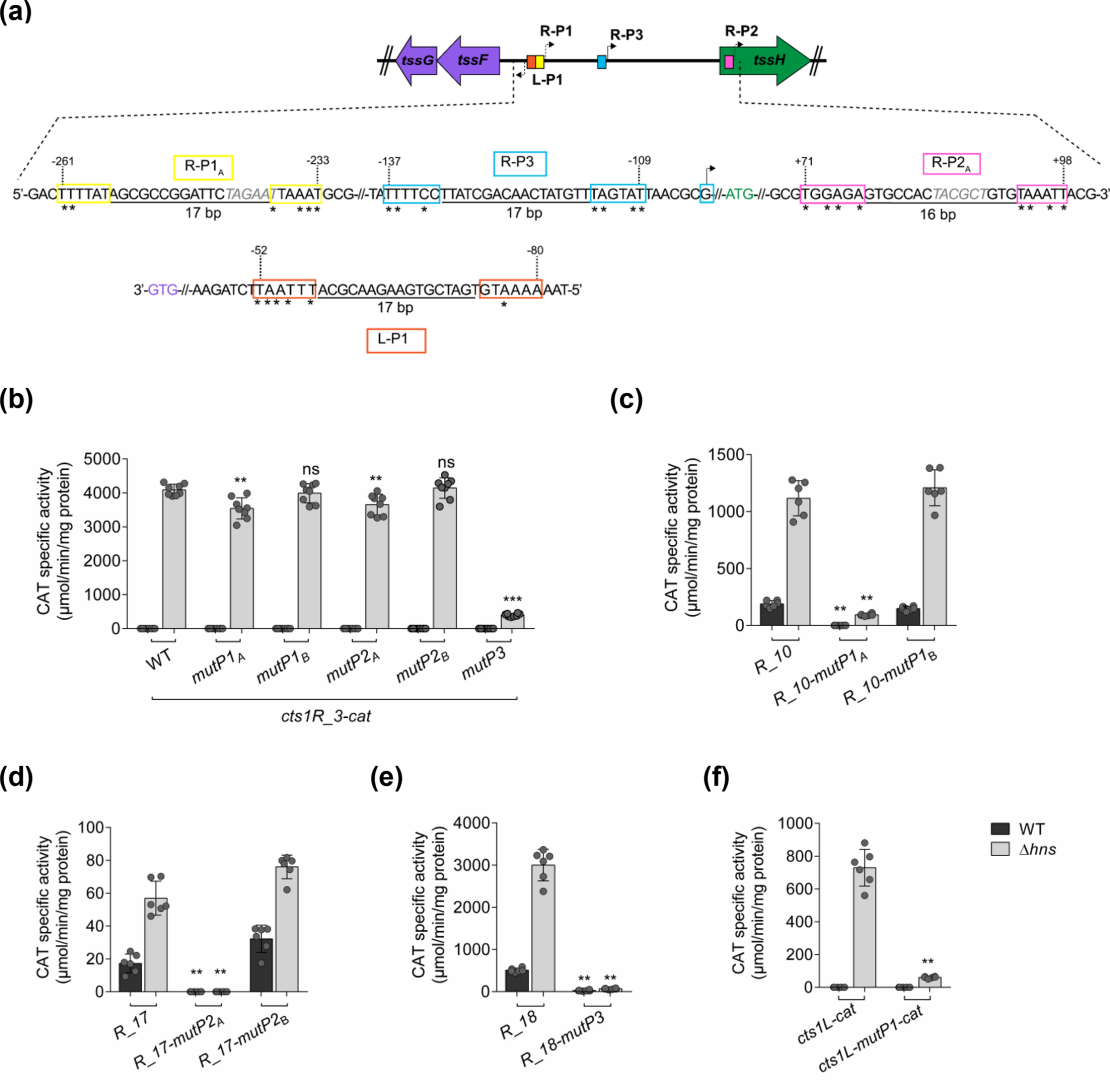
Mutational analysis defines four functional promoters in the *cts1* regulatory region. (**a**) Sequences of the *cts1* putative promoters. For each promoter, the functional −10 element – identified by mutational analysis and reanalysis against σ70 consensus features – is denoted with an ‘A’ subscript, whereas the alternative −10-like candidate (in some cases the original BPROM prediction), whose mutation did not affect basal activity, is denoted with a ‘B’ subscript. The R-P1_A_ region is located between positions −261 and −232; the R-P3 region is located between −137 and −109; and the R-P2_A_ region between +70 and + 98 relative to *tssH* start codon (indicated in green). Asterisks indicate σ70-conserved bases corresponding to the consensus −35 and −10 boxes (TTGACA and TATAAT, respectively). The TSS defined by 5′ RACE located 102 bases upstream of the *tssH* start codon and 7 bp downstream of the −10 element of R-P3 is illustrated in a blue box. The alternative −10 candidates (R-P1_B_ and R-P2_B_) are shown within the sequence in light grey italics. The l-P1 region (indicated in orange) is located between positions −79 and −51 bp relative to the *tssF* start codon (indicated in purple). l-P1 and R-P1_A_ regions are aligned to illustrate the proposed overlap of their −10 boxes. (**b–f**) Effect of −10 mutations on promoter activity. CAT-specific activity of the indicated transcriptional fusions and their −10 mutant derivatives was measured in *C. rodentium* WT and Δ*hns* strains as described in Fig. 1. (**b**) *cts1R_3-cat* carrying individual mutations in the P1_A_, P1_B_, P2_A_, P2_B_ or P3 −10 elements. (**c**) *cts1R_10-cat* (**R-P1**) with P1_A_ or P1_B_ mutations. (**d**) *cts1R_17-cat* (**R-P2**) with P2_A_ or P2_B_ mutations. (**e**) *cts1R_18-cat* (**R-P3**) with the P3 mutation. (**f**) *cts1L-cat* with the l-P1 mutation. Bars represent the mean±sd from at least three independent assays performed in duplicate. Statistical significance: ns *P*>0.05, *P≤0.05, **P≤0.01, ***P≤0.001, ****P≤0.0001.

To investigate the existence of the putative promoters, we first identified candidate −10 and −35 boxes using BPROM [40]. Because in silico prediction can be unreliable in AT-rich regions, we then reanalyzed these candidates against the features of functional σ70 promoters, paying particular attention to the conserved −10 positions (−11A and −7T), the -10/–35 spacing (optimally 17±1 bp) and the possible presence of UP elements, extended −10 motifs and discriminator regions [41, 42]. Based on this analysis, we selected for R-P1 and R-P2 two candidate −10 sequences each (designated A and B) and one for R-P3 (Fig. 4a). As described below, mutational analysis showed that the ‘A’ sequences are the functional −10 elements, whereas the ‘B’ sequences, although in some cases closer to the consensus, did not contribute to basal promoter activity.

To assess their functionality, we independently mutated the −10 elements within the *cts1R_3-cat* construct by replacing them with a SacI restriction site (GAGCTC), generating five new transcriptional fusions: *cts1R_3-*mutP1_A_-*cat, cts1R_3-*mutP1_B_-*cat, cts1R_3-*mutP2_A_-*cat, cts1R_3-*mutP2_B_-*cat* and *cts1R_3-*mutP3-*cat*. The activity of these fusions was assessed in WT *C. rodentium* and its ∆*hns* mutant (Fig. 4b).

The *cts1R_3-*mutP1_B_-*cat* and *cts1R_3-*mutP2_B_-*cat* constructs retained expression levels comparable to *cts1R_3-cat,* suggesting that these sequences do not contribute significantly to *cts1* expression under the tested conditions. In contrast, *cts1R_3-*mutP1_A_-*cat* and *cts1R_3-*mutP2_A_-*cat* exhibited reductions of approximately 14 and 11%, respectively, consistent with the dominance of P3 in this context; the contribution of P1 and P2 is much clearer in fusions lacking P3 (see below). The *cts1R_3-*mutP3-*cat* fusion showed an approximately 90% reduction in expression, confirming that P3 is the promoter responsible for most of the transcriptional activity under these conditions.

To further evaluate the role of R-P1, the same mutations were introduced into the *cts1R_10-cat* construct*,* which contains only this promoter*,* generating *cts1R_10-*mutP1_A_ and *cts1R_10-*mutP1_B._ The *cts1R_10-*mutP1_B_ fusion retained expression levels comparable to *cts1R_10,* whereas *cts1R_10-*mutP1_A_ showed an approximately 93% reduction in transcriptional activity relative to *cts1R_10-cat* (Fig. 4c), confirming the relevance of this sequence for *cts1* expression. Notably, for R-P1, the functional −10 element (P1_A_; TTAAAT) departs from the consensus, it lacks the −11A, whereas the consensus-closest candidate (P1_B_; TAGAAT, with −11A and −7T) was not functional. This illustrates that, in this AT-rich region, the sequence most similar to the consensus is not necessarily the active promoter, underscoring the need for experimental validation.

Similarly, mutation of P2_B_ in the *cts1R_17-cat* construct did not affect its expression levels (Fig. 4d). However, mutation of the P2_A_ −10 element completely abolished expression, confirming the effect of the functional P2 promoter (Fig. 4d).

Mutation of P3 in the context of *cts1R_18-cat* abolished expression in both WT and ∆*hns* strains (Fig. 4e). Using 5′ RACE, we mapped the TSS of R-P3 (Fig. 4a). On the basis of this experimentally defined TSS, we positioned the R-P3 −10 element as TAGTAT, which preserves the critical −11A and −7T and places the mapped +1 (a G) at the appropriate distance; the associated −35 region is weak (3/6 matches to the consensus) but is preceded by an AT-rich stretch that may act as an UP element. We were unable to determine the TSS for R-P1 or R-P2, probably because of their weaker expression.

To assess the functionality of the *cts1L* putative promoter, the −10 sequence of l-P1 within the *cts1L-cat* construct was mutated, generating the *cts1L-*mutP1-*cat* transcriptional fusion. This fusion exhibited an approximately 92% reduction in expression compared with *cts1L-cat*, confirming the functionality of the putative promoter (Fig. 4f). Notably, the proposed −10 box of l-P1 overlaps with that of R-P1A (Fig. 4a), suggesting that this region may function as an overlapping, bidirectional promoter element, although the precise boundaries of these −10 elements remain to be defined [43].

Together, these results support the presence of four promoters within the *cts1* regulatory region: one driving *cts1L* expression and three contributing to *cts1R* expression.

### CpxR contributes to *cts1* activation in the absence of H-NS, acting directly at internal promoters and indirectly at the divergent region

The CpxRA two-component system, composed of the sensor kinase CpxA and the response regulator CpxR, plays a central role in maintaining cell envelope integrity and modulating bacterial colonization and virulence, often in response to envelope stress [44].

Given that the response regulator CpxR was previously reported to modulate T6SS clusters [45], including genes located within the *cts1* cluster [46], we evaluated the effect of expressing it from a plasmid to better assess its potential role.

**Fig. 5.**
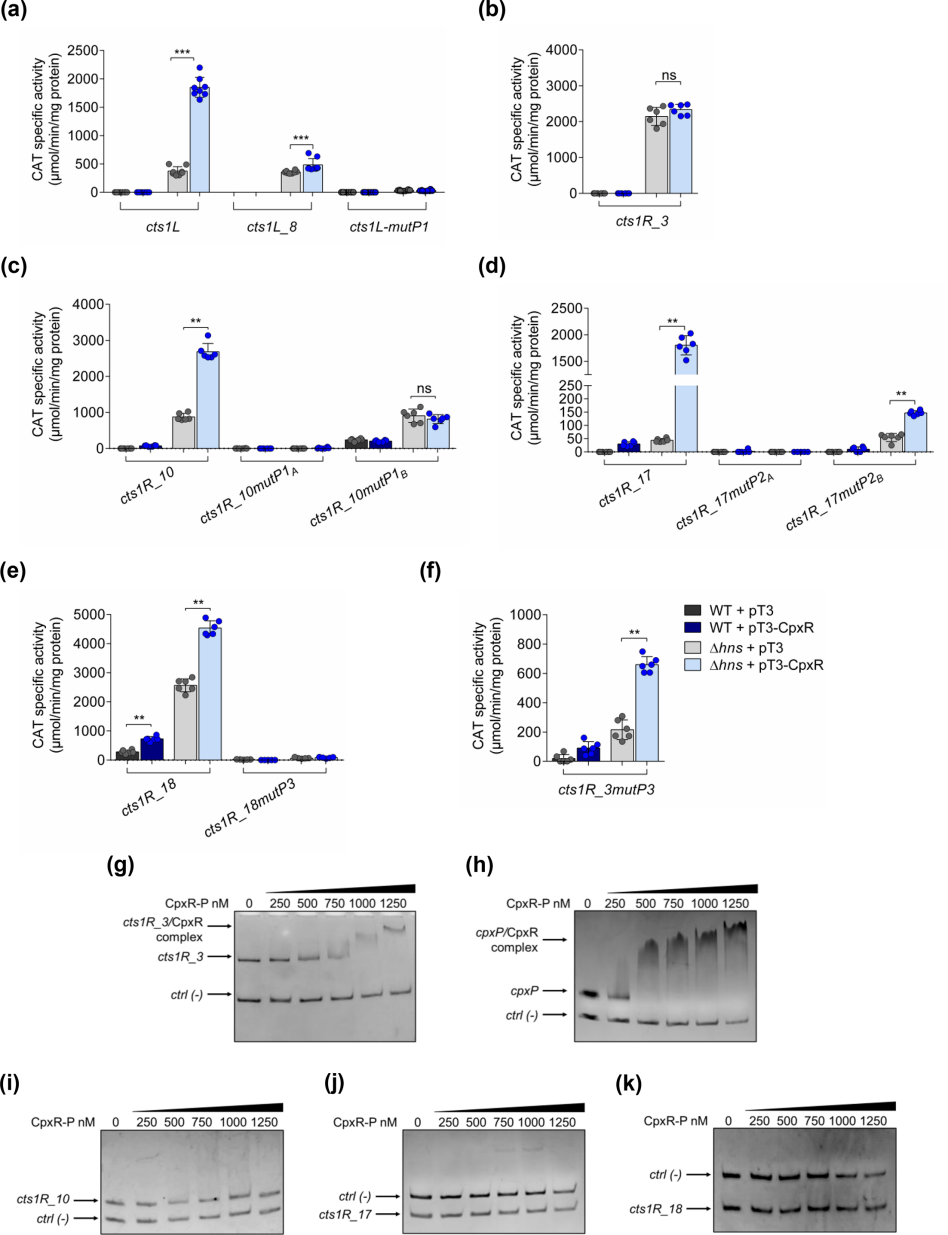
CpxR enhances *cts1* expression in the absence of H-NS and binds the internal but not the divergent promoters. **(a–f**) Effect of CpxR on *cts1* expression. CAT-specific activity was measured as described in Fig. 1. (**a**) *C. rodentium* carrying pT3-cpxR and *cts1L* transcriptional fusions *cts1L-cat*, *cts1L_8-cat* or *cts1L-mutP1-cat*. (**b**) *C. rodentium* carrying pT3-cpxR and *cts1R_3-cat*. (**c**) *C. rodentium* carrying pT3-cpxR and *cts1R_10-cat, cts1R_10mutP1_A_-cat* or *cts1R_10mutP1_B_-cat*. (**d**) *C. rodentium* carrying pT3-cpxR and *cts1R_17-cat, cts1R_17mutP2_A_-cat* or *cts1R_17mutP2_B_-cat*. (**e**) *C. rodentium* carrying pT3-cpxR and *cts1R_18-cat* or *cts1R_18mutP3-cat*. (**f**) *C. rodentium* carrying pT3-cpxR and *cts1R_3mutP-cat*. Bars represent the mean±sd from at least three independent assays performed in duplicate. Statistical significance was determined by a two-tailed Mann–Whitney U test: ns, *P*>0.05, *P≤0.05, **P≤0.01, ***P≤0.001, ****P≤0.0001. (**g–k**) EMSA of CpxR-P with *cts1* regulatory fragments. Fifty nanograms of the *cts1* (554 bp), *cpxP* (241 bp; positive control), *cts1R_10* (260 bp), *cts1R_17* (126 bp) or *cts1R_18* (114 bp) DNA fragments were incubated with increasing concentrations (0–1250 nM) of purified CpxR-P. An internal fragment of the *ROD_27801* coding region was used as a negative control (233 bp). Data shown are representative of three independent experiments that rendered similar results.

To evaluate the CpxR effect on *cts1L* expression, we introduced the pT3-CpxR (Table 1) into WT and ∆*hns C. rodentium* strains carrying the *cts1L-cat, cts1L_8-cat* and *cts1L_mutP1-cat* transcriptional fusions. The results revealed that CpxR significantly increased *cts1L-cat* expression in the ∆*hns* background (Fig. 5a), suggesting that it contributes to *cts1* activation once H-NS repression is relieved. No expression was detected for *cts1L_mutP1* in either condition, as expected given that this mutation abolishes the promoter itself. Notably, *cts1L_8-cat* showed a reduced activation by CpxR compared with *cts1L-cat,* indicating that the region between positions −420 to −112 contributes to CpxR-mediated activation.

CpxR expression had no detectable effect on *cts1R_3-cat* activity (Fig. 5b). To further investigate this result, we assessed the effect of CpxR on the individual activity of the R-P1, R-P2 and R-P3 promoters previously identified within the *cts1R* region.

In the absence of H-NS, expression levels of *cts1R_10-cat, cts1R_17-cat* and *cts1R_18-cat* were increased 8.3-, 40.9- and 1.7-fold, respectively, when CpxR was expressed from pT3-CpxR (Fig. 5c–e), with the strongest fold effect on *cts1R_17-cat* containing the P2_A_ promoter, reflecting its low basal activity rather than a high absolute expression level. These results suggest that the lack of an effect on overall *cts1R* expression when all three promoters are present likely reflects the dominance of P3 in the *cts1R_3-cat* construct, where P2_A_ contributes minimally to total transcription output.

No expression was detected for *cts1R_10mutP1_A_, cts1R_17mutP2_A_* or *cts1R_18mutP3*, confirming the functionality of the identified promoter regions (Fig. 5c–e). In contrast, *cts1R_10mutP1_B_* retained basal expression levels comparable to *cts1R_10*, but its activation by CpxR was reduced, suggesting that the P1_B_ sequence contributes to CpxR-dependent activation. Similarly, the increase in *cts1R_17mutP2_B_* expression in the presence of pT3-CpxR was lower than that observed for *cts1R_17*, suggesting that the P2_B_ sequence may also contribute to CpxR-dependent activation (Fig. 5d, e).

To further investigate why CpxR does not affect *cts1R* expression in the context of *cts1R_3*, we evaluated its effect on *cts1R_3mutP3* expression (Fig. 5f). We observed increased expression in the presence of pT3-CpxR; however, expression levels are low compared to the CpxR effect on *cts1R_17*. These results support the idea that CpxR acts primarily on the P1 and P2 promoters and that, when P3 is intact, it remains the dominant promoter under the tested conditions, masking the CpxR effect on P1 and P2.

We next used EMSAs to test whether CpxR binds the *cts1* divergent regulatory region directly. CpxR-P produced a concentration-dependent mobility shift of the fragment encompassing the *cts1L–cts1R* regulatory region (Fig. 5g); the *cpxP* regulatory region, a known CpxR target [38], served as a positive control (Fig. 5h) and a fragment from the *ROD_27801* coding region as a negative control. However, when we tested the individual P1, P2 and P3 promoter subfragments (*cts1R_10, cts1R_17* and *cts1R_18*), no CpxR-P binding was detected (Fig. 5i–k). Thus, although CpxR-P binds the extended divergent region, it does not bind the individual promoter regions whose activity responds to CpxR; the binding site, therefore, does not coincide with these promoters, consistent with an indirect effect on P1–P3.

Together, these findings indicate that while CpxR is not the factor responsible for relieving H-NS-mediated repression, it plays a key role in enhancing *cts1* expression once repression has been alleviated, likely by an as-yet unidentified element.

### Transcriptional organization of the *cts1R* cluster

Because large T6SS clusters often contain multiple transcriptional units and promoters, we analysed the transcriptional organization of the *cts1L* and *cts1R* gene clusters. We first used the Operon Mapper tool (https://biocomputo.ibt.unam.mx/operon_mapper/) [47] to predict putative internal transcriptional units. Because Operon Mapper infers operon structure from genomic context and functional associations between neighbouring genes rather than from promoter detection, we complemented these predictions with transcriptional fusions and in silico promoter searches to identify candidate internal promoters.

**Fig. 6.**
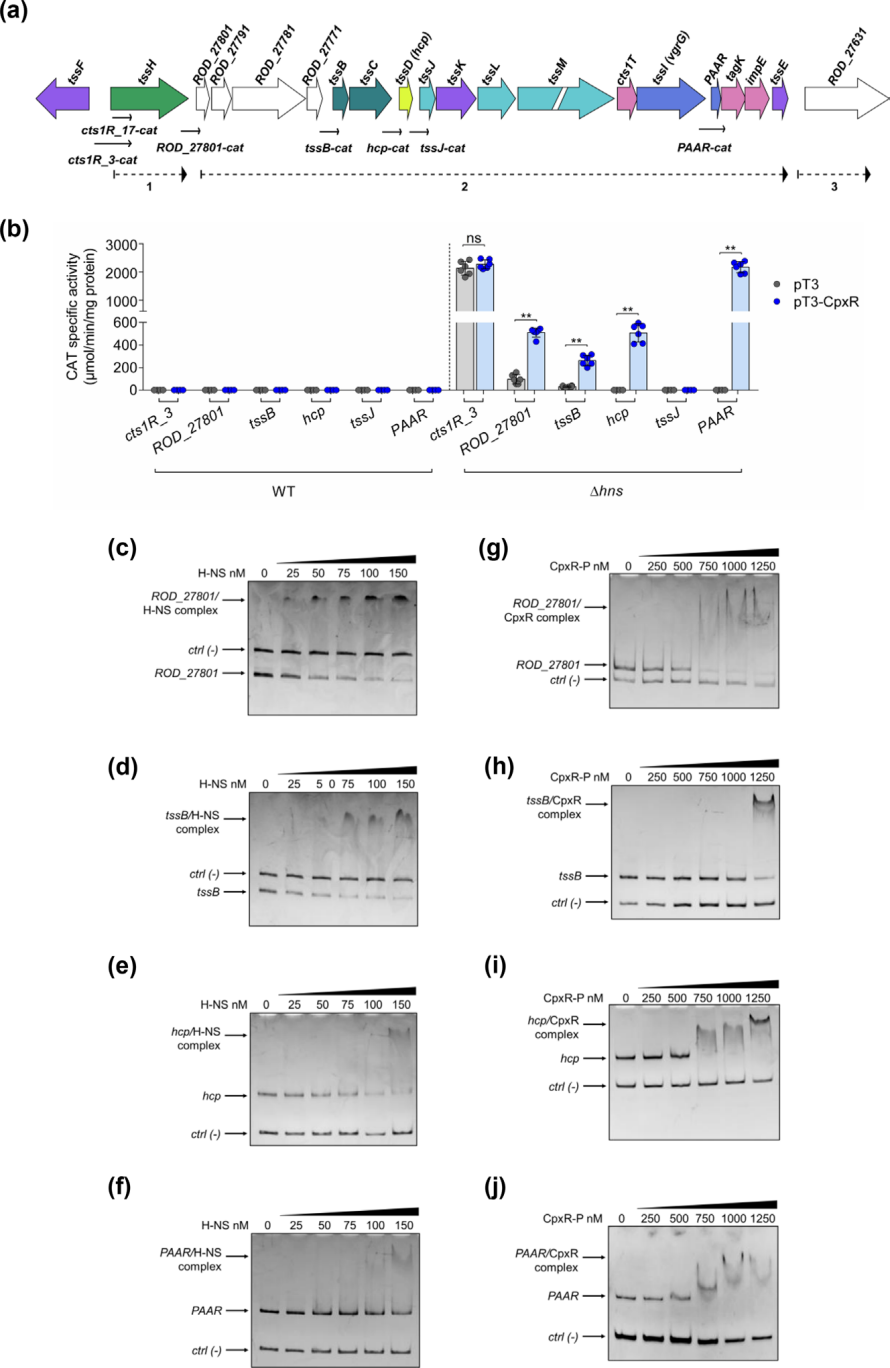
The *cts1R* cluster contains multiple internal promoters. (**a**) Schematic representation of the *cts1R* cluster, showing the transcriptional fusions used to identify internal promoters. Dashed arrows indicate the three transcriptional units predicted by Operon Mapper; the unit comprising *ROD_27631* was excluded from further analysis (see Results), as it is predicted to be transcribed independently and its product is unrelated to the T6SS. (**b**) Effect of H-NS and CpxR on the internal *cts1R* promoters. CAT-specific activity of the *ROD_27801*, *tssB, hcp* and *PAAR* transcriptional fusions was measured in *C. rodentium* WT and Δ*hns* strains, each in the absence or presence of the CpxR-expressing plasmid pT3-CpxR, as described in Fig. 1. Bars represent the mean±sd from three independent assays performed in duplicate. Statistical significance was determined by a two-tailed Mann–Whitney U test: ns *P*>0.05, *P≤0.05, **P≤0.01, ***P≤0.001, ****P≤0.0001. (**c–f**) EMSA showing H-NS binding. Fifty ng of the *ROD_27801* promoter region (300 bp), tssB (332 bp), *hcp* (441 bp) or PAAR (506 bp) DNA fragments were incubated with increasing concentrations (0–150 nM) of purified H-NS. A larger internal fragment of *ROD_27801* coding sequence was used as a negative control (471 bp). Data shown are representative of at least three independent experiments that rendered similar results. (**g–j**) EMSA showing CpxR-P binding. Fifty ng of the *ROD_27801* (300 bp), *tssB* (332 bp), *hcp* (441 bp) or *PAAR* (506 bp) DNA fragments were incubated with increasing concentrations (0–1250 nM) of purified CpxR-P. An internal fragment of the *ROD_27801* coding region was used as a negative control (233 bp). Data shown are representative of at least three independent experiments that rendered similar results.

According to this analysis, the *cts1R* cluster was predicted to comprise three transcriptional units: the first encoding *tssH*, expressed from the divergent regulatory region; the second comprising 17 genes from *ROD_27801* to *tssE*; and a third consisting solely of *ROD_27631* (Fig. 6a). Because *ROD_27631* is predicted to form its own transcriptional unit, separate from the main T6SS operon and because its putative product (a predicted autotransporter) has no reported association with the T6SS, we concluded that it is transcribed independently from the *cts1R* cluster and excluded it from further analyses.

Because the second predicted transcriptional unit encompasses 17 genes and several of which are separated by intergenic regions longer than 50 bp, we hypothesized that additional internal promoters may facilitate adequate expression across the *cts1R* cluster. To test this possibility, we constructed *cat* transcriptional fusions for several intergenic regions within the cluster (Fig. 6a) and evaluated their activity in WT and Δ*hns C. rodentium* strains carrying pT3-CpxR (Fig. 6b).

Our results revealed the presence of functional promoters upstream of *ROD_27801, tssB, hcp* and *PAAR*. In the WT strain, CpxR had no effect on promoter activity and expression remained repressed. In contrast, in the Δ*hns* strain, expression from the *ROD_27801* and *tssB* promoter regions increased 16.4- and 31.8-fold, respectively, indicating that the positive effect of CpxR on *cts1* promoter activation depends on the absence of H-NS. Notably, *hcp* and *PAAR*, which showed no detectable activity in the Δ*hns* strain alone, were activated by CpxR from this undetectable basal level, indicating that relief of H-NS repression is necessary but not sufficient for their activation.

In silico analysis of the intergenic regions between *ROD_27771-tssB*, *tssC-hcp, hcp-tssJ* and *vgrG-PAAR* suggested the presence of putative promoters (Fig. S2); however, additional experiments will be required to confirm their functionality.

To test whether H-NS and CpxR act directly at these internal promoters, we performed EMSAs with the corresponding promoter fragments. H-NS bound all four regions (Fig. 6c–f) and, in contrast to the divergent P1–P3 promoters, CpxR-P also bound them directly (Fig. 6g–j). Thus, CpxR regulates the internal *cts1R* promoters directly, whereas its effect on the divergent P1–P3 promoters is indirect.

Together, these findings suggest that the *cts1R* cluster contains multiple internal promoters regulated by H-NS and CpxR and is likely organized into more than three transcriptional units.

### Transcriptional organization of the *cts1L* cluster

For the *cts1L* cluster, its seven genes were predicted to be transcribed as a single unit (Fig. S3A). To test this prediction, we performed reverse transcription PCR (RT-PCR) analysis using RNA isolated from *C. rodentium* Δ*hns*. cDNA synthesis was carried out with a reverse primer annealing to the last gene of the cluster*, cts1W* (Table 2). All primer pairs targeting the intergenic regions between *cts1W* and *cts1O*, *ROD_27861* and *ROD_27851, ROD_27851* and *tssA* and *tssG* and *tssF* produced amplicons (Fig. S3B). Because cDNA was synthesized with a reverse primer annealing to the most distal gene, *cts1W*, the detection of these upstream contiguous pairs indicates that the *cts1L* genes lie on a continuous mRNA extending at least to *cts1W*, consistent with co-transcription across the cluster. As a negative control, no amplification was observed for the unrelated *gcfA* gene, which encodes a fimbrial subunit located outside of the *cts1L* cluster [36]. Unlike for *cts1R*, the operon prediction by Operon Mapper for *cts1L* is consistent with the short or overlapping intergenic regions within the cluster (*tssF–tssG, tssG–tssA* and *ROD_27861–cts1O* overlap; *tssA–ROD_27851*, 3 bp; *ROD_27851–ROD_27861*, 25 bp; *cts1O–cts1W*, 25 bp). A BPROM search of these regions identified only a few candidates at positions that do not suggest functional internal promoters; however, given the limited reliability of in silico prediction in these regions (as noted for *cts1R*), internal promoters cannot be formally excluded.

Taken together, these data indicate that the *cts1L* genes are co-transcribed on a transcript extending at least to *cts1W*, but do not formally establish a single transcriptional unit; distinguishing this from the presence of additional internal promoters will require further analysis, such as northern blotting or amplification of a full-length product.

## Discussion

**Fig. 7.**
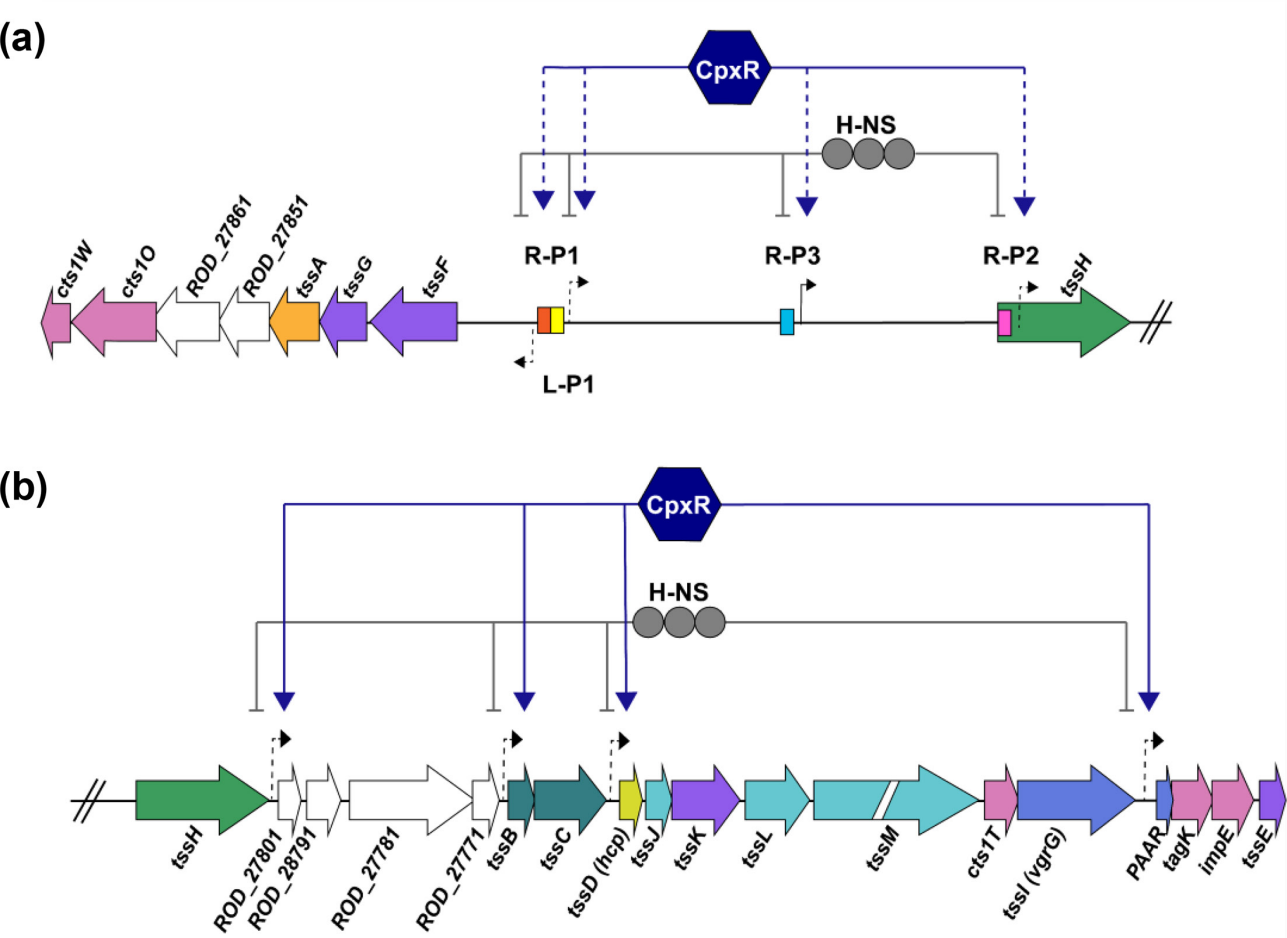
Proposed model of *cts1* regulation. **(a**) Regulation of the *cts1L–cts1R* divergent regulatory region. The *cts1L* promoter l-P1 (orange) and the *cts1R* promoters R-P1 (yellow), R-P3 (blue) and R-P2 (pink; located within the *tssH* coding sequence) are silenced by H-NS, which binds the regulatory region directly. RT-PCR indicates that the *cts1L* genes are co-transcribed on a transcript extending to *cts1W*. Upon relief of H-NS repression, these divergent promoters are activated in a CpxR-dependent manner; because CpxR-P does not bind these promoter regions *in vitro* (it shifts the larger divergent fragment but not the individual P1–P3 subfragments), this activation is depicted as indirect (dashed arrows), mediated by an as-yet-unidentified regulator. (**b**) Regulation of the internal *cts1R* promoters. Additional promoters within the *cts1R* cluster (P*_ROD_27801_*, P*_tssB_*, P*_hcp_* and P*_PAAR_*) drive expression of downstream genes. These promoters are silenced by H-NS and, in contrast to the divergent promoters, are bound and activated directly by CpxR-P. H-NS molecules are shown as grey circles and CpxR-P molecules as dark blue hexagons. Solid arrows indicate direct interactions demonstrated by EMSA (H-NS with the divergent and internal regions; CpxR-P with the internal promoters); dashed arrows indicate indirect or proposed regulatory interactions that remain to be experimentally confirmed (CpxR-dependent activation of the divergent promoters via the unidentified regulator). Bent arrows indicate TSSs; all are putative except R-P3, which was mapped by 5′ RACE.

In this study, we provide new insights into the regulatory mechanism governing the *cts1* locus of *C. rodentium* (Fig. 7). Previous studies demonstrated that expression of both the *cts1L* and *cts1R* clusters is required for Hcp secretion and interbacterial competition [9] and that a T6SS-1-deficient strain displays impaired intestinal colonization in a murine host [20]. However, the *cts1* locus is not transcriptionally active under routine laboratory growth conditions.

We demonstrated that the *cts1* locus is strongly repressed by the NAP H-NS. H-NS-mediated silencing of T6SS clusters has been reported in a variety of bacteria [15, 48–59]. The prominent role of H-NS in repressing these clusters likely reflects their frequent association with horizontally acquired genomic regions and their elevated A-T content [5, 30, 60, 61]. Although the overall A-T content of the *cts1* locus (46%) is similar to that of the *C. rodentium* genome (45.3%) [26], several intergenic regions analysed here displayed elevated A-T content (*cts1L–R*, 60%; *ROD_27801*, 64%; *tssB*, 50%; *hcp*, 71%; *PAAR*, 49%), consistent with preferential targeting by H-NS.

More broadly, H-NS has been proposed to act as a global suppressor of pervasive transcription by silencing promoter-like sequences that frequently arise in AT-rich DNA [62, 63]. In this context, the transcriptional inactivity of *cts1* under laboratory conditions likely reflects H-NS-dependent xenogeneic silencing, which prevents spurious transcription from multiple potential promoters embedded within the locus. Overcoming this repression generally requires specific environmental or regulatory signals. For instance, in *V. parahaemolyticus*, surface sensing and high-salt conditions relieve H-NS repression [8], whereas in *Edwardsiella piscicida*, the regulator EnrR derepresses *esrB,* a gene encoding a key T6SS activator, by competing with H-NS binding [64].

Using a systematic approach based on sequential deletions and transcriptional fusions, we dissected the *cts1L-cts1R* divergent regulatory region and identified multiple promoter elements, including one directing transcription towards *cts1L,* three towards *cts1R* and several additional internal promoters within the *cts1R* cluster. Rather than representing a set of independently regulated promoters, these elements may reflect a promoter-rich regulatory architecture in which the functional contribution of individual promoters depends on DNA context, regulatory protein binding and environmental conditions.

Our results also highlight the challenges of defining functional promoters in isolation. Differences in fragment length and context may influence promoter output by altering local DNA structure, RNA stability or transcriptional dynamics [39, 65]. This consideration applies specifically to fusions that share a promoter but differ in the sequence located between the promoter and the *cat* reporter: where the downstream sequence differs, such RNA-level effects cannot be excluded and may contribute to the differences in expression. Importantly, however, this is not the case for all of our comparisons. For fusions that differ only in their upstream sequence while sharing the same 3′ end, for example, *cts1L_8* and *cts1L_1*, any RNA-level effect on the transcribed region would be identical in both, so it cannot account for the difference in expression; the reduced activity of *cts1L_8,* therefore, reflects the loss of a 5′ element acting upstream of the promoter. In addition, the promoter activity detected in a *Δhns* mutant does not by itself establish the presence of dedicated promoters: in AT-rich regions, relief of H-NS silencing can give rise to spurious transcription from promoter-like sequences that may not be functionally relevant under physiological conditions [31, 55, 66]. To minimize this limitation, we evaluated two different −10 sequences for each putative promoter and observed that not all A-T-rich sequences behaved as functional promoters in a *Δhns* mutant. Nevertheless, additional experiments will be required to confirm the functionality of l-P1, R-P1 and R-P2, as well as of the internal putative promoters within the *cts1R* cluster.

Notably, the proposed −10 elements of l-P1 and R-P1 appear to overlap, although their precise boundaries remain to be defined. Traditionally, overlapping promoters were considered mutually exclusive due to competition between RNA polymerases binding at adjacent promoters [67, 68]. However, more recent studies suggest that transcription can occur in both directions, albeit with different efficiencies [43]. In the context of *cts1,* this architecture may provide flexibility in the regulation of divergent gene clusters. However, the coordination of transcription from these overlapping promoters remains to be experimentally addressed, particularly considering that expression of both *cts1L* and *cts1R* is required for CTS1 functionality [9].

In addition to H-NS, our results indicate that the response regulator CpxR influences *cts1* expression. CpxR enhanced the activity of several promoters in the absence of H-NS, including both the intergenic divergent promoters and the internal *cts1R* promoters. Its effects were not uniform across constructs, however, and no clear activation was observed when the complete *cts1L–cts1R* regulatory region (*cts1R_3*) was analysed. Notably, for the *hcp* and *PAAR* promoters, relief of H-NS repression was necessary but not sufficient for activation, which additionally required CpxR. These observations indicate that CpxR does not act through a single dominant promoter but contributes to *cts1* expression at multiple promoters in a context-dependent manner.

The mechanism, however, differs between the two groups of promoters. In EMSA, CpxR-P bound the internal *cts1R* promoter fragments directly but did not bind the individual P1, P2 or P3 promoter subfragments, even though it shifted the larger divergent *cts1R_3* fragment. The simplest interpretation is that CpxR acts directly at the internal promoters but indirectly at the divergent P1–P3 promoters, most likely through an as-yet-unidentified regulator whose action depends on prior relief of H-NS repression.

Consistent with this, no canonical CpxR-binding motif was found within the *cts1L–cts1R* region or at the internal promoters, even though CpxR-P bound the latter directly. This is not unexpected, as CpxR can bind promoters that lack obvious consensus sequences, tolerating substantial sequence degeneracy or relying on broader promoter architecture rather than strictly conserved motifs [69–75]. At the divergent region, the CpxR-P shift of the larger *cts1R_3* fragment, absent with the smaller P1–P3 subfragments, may, therefore, reflect a low-affinity or context-dependent interaction at a site that does not coincide with these promoters. The location of this site, and the identity of the intermediate regulator, remain to be established.

The differential responsiveness of these promoters to CpxR raises the possibility that *cts1* expression is deployed in a stepwise manner, with individual promoters showing different sensitivities to regulatory inputs and allowing subsets of genes to be activated under distinct conditions or levels of regulatory activity.

From a physiological perspective, activation of *cts1* is likely to occur under conditions encountered during host colonization. Although we were unable to identify laboratory conditions that fully relieve H-NS repression, growth under 5% CO_2_ in minimal medium led to increased expression, suggesting that specific environmental signals may partially activate the system. Given that the Cpx system responds to multiple envelope-associated and environmental stresses [76–82], it is plausible that *cts1* expression is coordinated with environmental cues encountered in the intestinal niche.

This idea is consistent with previous observations showing that the Cpx pathway contributes to *C. rodentium* colonization, as deletion of *cpxRA* impairs colonization of the murine host, likely as a consequence of impaired growth within the colonic environment resulting from the combined effects of several Cpx-regulated genes, including those involved in virulence [46, 83–85]. CpxRA activity appears to be particularly important during the early stages of infection, when its expression peaks and subsequently declines as colonization progresses [83], consistent with the role of CTS1 in promoting competition against the murine microbiota during the onset of infection [20].

Although the specific signals that activate the Cpx pathway to induce *cts1* expression remain unknown, CpxA has been reported to sense indole, a microbiota-derived signalling molecule [86]. This raises the possibility of a crosstalk between the microbiota and the Cpx system that could converge on *cts1* regulation. However, because CpxR requires prior relief of H-NS-mediated repression, it is unlikely that the same signal simultaneously activates the Cpx pathway and directly triggers *cts1* expression.

Together, our findings support a model in which the *cts1* locus functions as a highly regulated promoter-rich region whose expression emerges from the interplay between environmental signals, H-NS-mediated repression, promoter architecture and CpxR-dependent enhancement upon derepression. Future studies aimed at identifying the signals that relieve H-NS silencing, defining the intermediate regulator and other regulatory elements and determining promoter usage during infection will be essential to understand how this T6SS is regulated *in vivo*.

## Supplementary material

10.1099/mic.0.001756 Supplementary Material 1.

## Abbreviations

CAT: chloramphenicol acetyltransferase
CpxR-P: phosphorylated CpxR
DMEM: Dulbecco’s Modified Eagle's Medium
EMSA: electrophoretic mobility shift assay
LB: Luria-Bertani
MM-N: Minimal Medium N
NAP: nucleoid-associated protein
5′ RACE: 5′ rapid amplification of cDNA end
RT-PCR: reverse transcription PCR
TSS: transcription start site
T6SS: type VI secretion system
