# Supplementary material for: The divergent cts1 type VI secretion system locus of Citrobacter rodentium is controlled by multiple promoters that are silenced by H-NS, with CpxR contributing to their activation upon derepression
Source: Microbiology (Reading). 2026 Aug 12;172(8):001756. doi: 10.1099/mic.0.001756 (PMC13475847; doi:10.1099/mic.0.001756)
Supplement: Supplementary file 1 [file mic-172-01756-s001.pdf]

Media	Temperature ºC	Atmosphere
LB	25	Shaking
	30	Shaking
	37	Shaking
		Statics
		Statics + 5%CO_2
LB + agar	37	Statics
DMEM	25	Shaking
	30	Shaking
	37	Shaking
		Statics
		Statics + 5%CO_2
DMEM (HeLa cells supernatant)	37	Statics + 5%CO_2
DMEM (Caco-2 cells supernatant)	37	Statics + 5%CO_2
McCoy's 5A (HT-29 cells supernatant)	37	Statics + 5%CO_2
MM-$N_{glucose}$	25	Shaking
	30	Shaking
	37	Shaking
		Statics
		Statics + 5%CO_2
MM-$N_{glucose}$ + agar	37	Statics
MM-$N_{glycerol}$	37	Statics + 5%CO_2

Supplementary Table 1. Experimental conditions used to evaluate *cts1* expression.

Expression of *cts1L-cat* and *cts1R-cat* was assessed in *C. rodentium* WT.

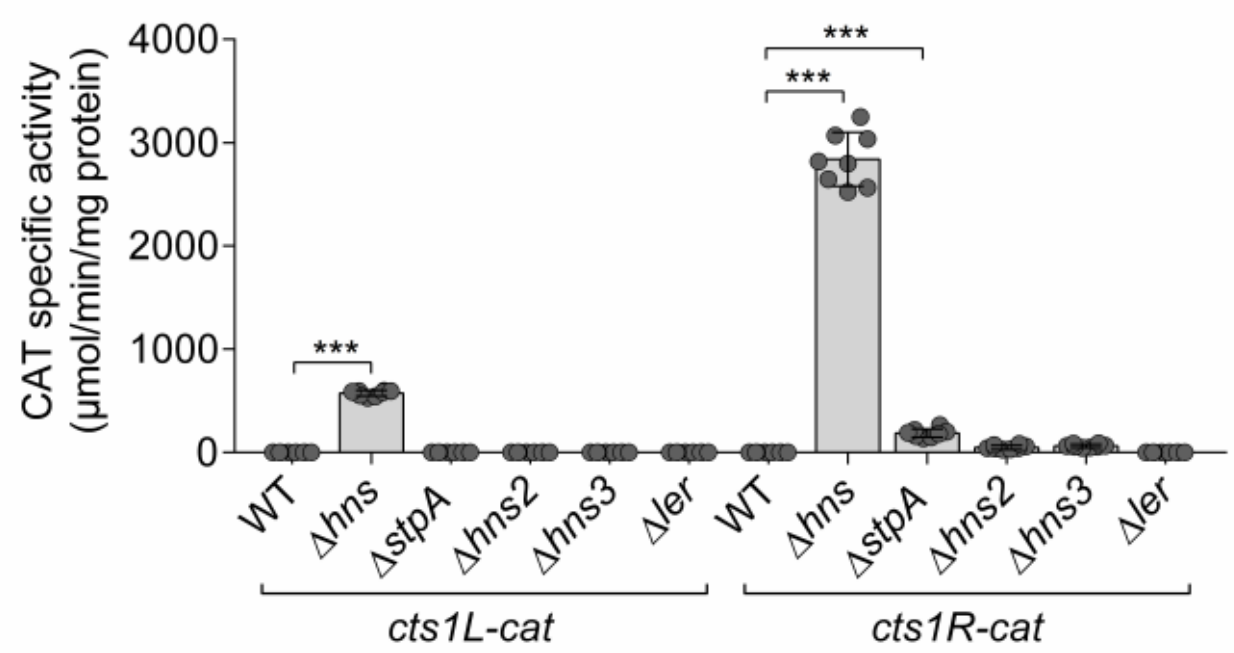

Supplementary Figure 1. H-NS is the primary repressor of *cts1* expression.

Expression of the *cts1L-cat* and *cts1R-cat* transcriptional fusions was evaluated in *C. rodentium* WT and mutants lacking the H-NS paralogues Δ*hns,* Δ*stpA*, Δ*hns2*, Δ*hns3*, and Δ*ler* strains (Table 1). CAT-specific activity was measured as described in Figure 1. Bars represent the mean ± SD from four independent assays, each performed in duplicate. Statistical significance was determined by a two-tailed Mann-Whitney U test: ns $P > 0.05$, * $P \leq 0.05$, ** $P \leq 0.01$, *** $P \leq 0.001$, **** $P \leq 0.0001$.

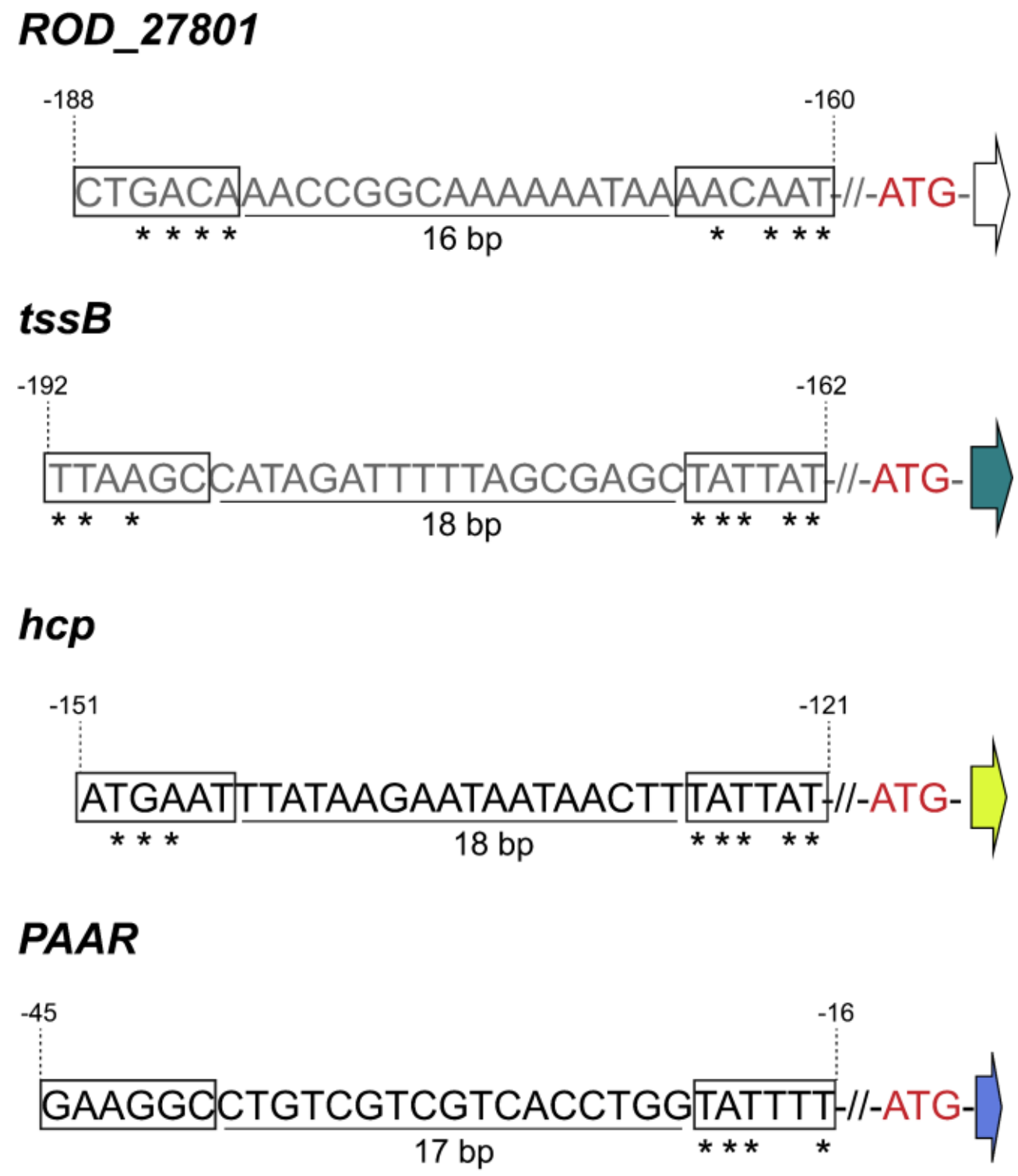

Supplementary Figure 2. Prediction of internal promoters within the *cts1R* cluster.

Sequences of putative *cts1R* internal promoters for the *ROD_27801, tssB, hcp,* and *PAAR* genes. Candidate -10 elements were identified using BPROM and reanalyzed against σ70 consensus features (-11A, -7T, and -10/-35 spacing). Coordinates of each promoter region are given relative to the corresponding ATG start codon (indicated in red). Asterisks indicate σ70-conserved bases corresponding to the consensus -35 and -10 boxes (TTGACA and TATAAT, respectively). Sequences illustrated in gray indicate that the promoter is located in the structural region of the upstream gene, while sequences in black indicate promoters located in the intergenic region.

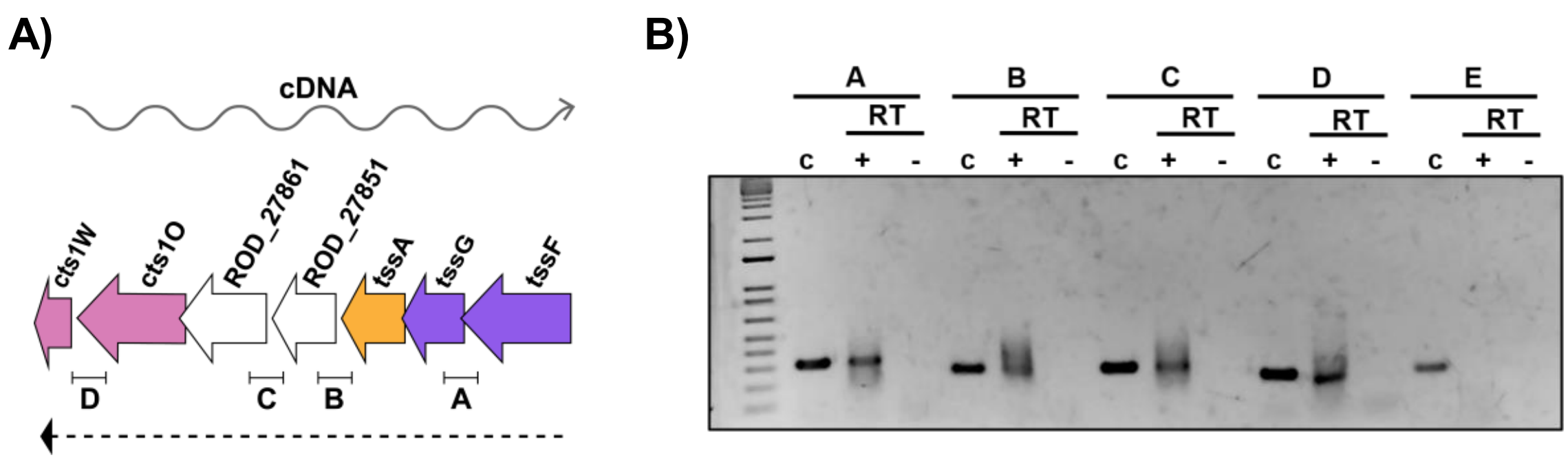

Supplementary Figure 3. Transcriptional organization of the *cts1L* cluster.

A) Operon Mapper predicted that *cts1L* is transcribed as a single transcriptional unit. Schematic representation of the *cts1L* operon showing the four intergenic regions evaluated by RT-PCR: A (*cts1O-W*), B (*ROD_27851-27861*), C (*tssA-ROD_27851*), and D (*tssF-G*). cDNA was synthesized using the *cts1O-cts1W* reverse primer (Table 2), which anneals to *cts1W*.

B) RT-PCR analysis. RNA was extracted from *C. rodentium* Δ*hns* grown in MM-N for 12 h. at 37 ºC under 5% CO_2 atmosphere. RT-PCR reactions were carried out with (+) or without (-) reverse transcriptase; c, control PCR using genomic DNA from *C. rodentium* as template. A *gcfA* fragment (labeled E), from a gene outside the *cts1L* cluster, served as a negative control.
